# Safety and Efficacy of Botulinum Toxin to Preserve Gland Function after Radiotherapy in Patients with Head and Neck Cancer: A Prospective, Randomized, Placebo-Controlled, Double-Blinded Phase I Clinical Trial

**DOI:** 10.1371/journal.pone.0151316

**Published:** 2016-03-18

**Authors:** Afshin Teymoortash, Andreas Pfestroff, Andrea Wittig, Nora Franke, Stephan Hoch, Susanne Harnisch, Carmen Schade-Brittinger, Helmut Hoeffken, Rita Engenhart-Cabillic, Markus Brugger, Konstantin Strauch

**Affiliations:** 1 Department of Otolaryngology, Head and Neck Surgery, Philipp University, Marburg, Germany; 2 Department of Nuclear Medicine, Philipp University, Marburg, Germany; 3 Department of Radiotherapy and Radio-Oncology, Philipp University, Marburg, Germany; 4 Center for Clinical Trials, Philipp University, Marburg, Germany; 5 Institute of Medical Informatics, Biometry and Epidemiology, Chair of Genetic Epidemiology, Ludwig-Maximilians-Universität, Munich, Germany; 6 Institute of Genetic Epidemiology, Helmholtz Zentrum München—German Research Center for Environmental Health, Neuherberg, Germany; 7 Institute of Medical Biometry and Epidemiology, Philipp University, Marburg, Germany; Cardiff University, UNITED KINGDOM

## Abstract

This prospective, randomized, placebo-controlled, double-blinded phase I clinical trial investigates safety and efficacy of botulinum toxin (BoNT) to preserve gland function after radiotherapy in patients with head and neck cancer. Twelve patients with advanced head and neck cancer were injected with BoNT into the submandibular glands prior to primary radiochemotherapy. Six patients received BoNT/A and 6 patients BoNT/A and B, half of each subgroup into their left and the other half into their right gland. As an internal control, sodium chloride was injected into the respective contralateral gland (placebo). For the evaluation of the salivary gland function, technetium pertechnetate salivary gland scintigraphy was performed before and after the end of radiotherapy. BoNT/A and B were well tolerated. Analysis of the scintigraphic data revealed no statistically significant difference between BoNT and placebo regarding the scintigraphic uptake difference (pBoNT/A = 0.84 and pBoNT/A-B = 0.56 for BoNT/A vs. placebo and BoNT/A-B vs. placebo, respectively). We also found no significant difference in treatment between BoNT and placebo in terms of salivary excretion fraction (pBoNT/A = 0.44; pBoNT/A-B = 0.44). This study demonstrates that BoNT can be safely combined with radiochemotherapy. Dosing and timing of BoNT injection should be further investigated for efficacy analysis.

**Trial Registration:**

German Registry for Clinical Trails DRKS00004595

## Introduction

Radiotherapy plays a central role in the treatment of advanced squamous cell carcinoma of the head and neck region (HNSCC). Radiotherapy in curative intent conventionally involves the administration of a total dose of 60–70 Gy in daily fractions of 1.8–2 Gy over 6–7 weeks. Locoregional control might be further improved by hyperfractionated and/or accelerated radiotherapy regimes (HART) in comparison to the conventional fractionation. Chemotherapy combinations such as fluorouracil and cisplatin, given concurrently with radiation, have demonstrated improvements in both locoregional control and survival [1,2].

A clinically very relevant side effect of radiotherapy to the head and neck region, however, is damage of the salivary glands. Radiation doses as necessary for curative treatments of HNSCC lead to a significant and often permanent decrease of salivary flow [3]. Radiation-induced sialadenitis can cause dysphagia, impaired sense of taste, infectious diseases of the oral and pharyngeal mucosa, and dental and periodontal disease. Such salivary gland dysfunction, associated with irreversible xerostomia, is a very serious clinical problem, which significantly impairs quality of life [4].

Because of the high clinical relevance of radiation-induced sialadenitis, research is ongoing to develop and test radioprotective agents for salivary glands. Despite many attempts and efforts in studies on human salivary glands and in animal models, relevant progress in preventing or treating irradiation-induced sialadenitis could not be achieved so far. Some preventive interventions were reported in the literature including salivary gland transfer and application of cytoprotectants, such as amifostine, and pilocarpine as a parasympathomimetic agent administered during radiation therapy [5]. These studies, however, have found varied success rates with a significant proportion of patients who continue to experience xerostomia and who suffer from potential adverse effects [6,7]. Thus none of those approaches are established in clinical setting.

Botulinum toxin (BoNT) is an established agent in the treatment of different neurologic disorders and is increasingly used for the treatment of salivary gland diseases such as sialorrhea [8]. Recently, we could show in a preliminary study in rats that intraglandular injection of BoNT performed prior to radiation significantly reduced radiation-induced toxicity of the glandular tissue [9]. The aim of the present study was therefore to analyze safety and efficacy of BoNT, injected into the submandibular glands prior to radiochemotherapy for HNSCC to preserve the gland function.

## Patients and Methods

### Study design and quality assurance

The study was designed as a four-armed randomized, placebo-controlled, double-blinded single-center study (German Clinical Trials Register ID: DRKS00004595; European Union Drug Regulating Authorities Clinical Trials (EudraCT) No.: 2009-014576-22; Federal Institute for Drugs and Medical Devices (BfArM) No.: 4035548). The primary objectives of the study were safety, tolerability, and effect of BoNT in preserving salivary gland function of patients with HNSCC after radiotherapy. The study was carried out in accordance with good clinical practice guidelines, national laws, and the Declaration of Helsinki in the Department of Otolaryngology, Head and Neck Surgery, Philipp University, Marburg, Germany. The study was reviewed and approved by the Ethics Committee of the medical faculty, Philipp University, on 11. September 2009 (No: 115/09). All patients gave written informed consent prior to their inclusion. Quality control included data source verification by monitoring, double data entry and in-house monitoring in the central study office. A trial description can be found on the website of the German Clinical Trials Registry (DRKS): https://drks-neu.uniklinik-freiburg.de/drks_web/navigate.do?navigationId=trial.HTML&TRIAL_ID=DRKS00004595. The study registration was performed in delay after enrolment of the first patient. The authors confirm that all ongoing and related trials for the study drug are registered.

### Patient selection

Due to the phase I nature of the study, the number of recruited patients was kept to a minimum. Twelve patients with advanced HNSCC were aimed to be recruited to the study. Depending on the true effect size, this phase I clinical trial with a limited number of patients might not have sufficient power to detect a treatment effect of BoNT (for more details about the power calculation please see subsection statistical analysis of scintigraphic data). Patients were included in the study only if they had normal and symmetric salivary gland function in salivary gland scintigraphy at baseline examination prior to BoNT injection and prior to radiochemotherapy. Exclusion criteria were: extirpation of the submandibular gland, diseases of the salivary gland, medication that might influence the salivary function, thyreostatic drugs, previous radiotherapy and pregnancy. Patients were recruited between July 2010 and October 2011 at the Department of Otolaryngology, Head and Neck Surgery, Philipp University, Marburg, Germany. The trial ended in October 2012 after the last patient had been examined in the follow-up.

### Radiochemotherapy

Protocol treatment included radiochemotherapy in a hyperfractionated accelerated regime, performed in an intensity modulated technique (IMRT) to a total absorbed dose of 72 Gy (HART-B). Simultaneous chemotherapy with 5-fluoruracil (5FU) (600 mg/m^2^, day 1–5) and cisplatin (once weekly, 30 mg/m^2^) was scheduled for all patients. The described tumor spread requires irradiation of a target volume that includes the submandibular region without the possibility to spare the submandibular glands.

### Treatment with botulinum toxin and randomization

All patients were treated either with 15 U BoNT/A (Allergan Pharmaceuticals, Ireland) or 15 U BoNT/A and 750 U BoNT/B (Eisai Manufacturing Knowledge Centre, United Kingdom). The treatment with a mixture of BoNT/A and B was based on the results of our previous study, with the most prominent morphological effect after the combined injection of BoNT/A and B [10]. A randomization list was created prior to recruitment of patients to contain a random sequence of length 12 with assignments 1, 2, 3, and 4, such that each of the four assignments occurred three times, corresponding to three patients per subgroup. This list was created by the responsible biometrician (KS) prior to patient recruitment, and the assignment of patients to treatments was done by the Coordination Center for Clinical Trials according to this randomization list. Patients were randomized into one of the following 4 treatment groups:

1. BoNT/A injected in the right gland, NaCl (0.9%) in the left gland;
2. BoNT/A in the left gland, NaCl (0.9%) in the right gland;
3. BoNT/A and B in the right gland, NaCl (0.9%) in the left gland;
4. BoNT/A and B in the left gland, NaCl (0.9%) in the right gland.

This procedure resulted in a balanced allocation of 3 patients to each of the 4 treatment groups so that 6 patients received either BoNT/A or BoNT/A and B each. The BoNT or NaCl was injected into the submandibular glands by ultrasound guidance 14–21 days before starting radiochemotherapy. The choice of this time interval was based on the effects seen in rats after radiotherapy [9]. In addition, the reduction of salivary flow with BoNT for treatment of hypersalivation reaches its full extent 2–3 weeks after intraglandular injection [11].

To achieve double-blindness, the medication was provided to the clinician with coded numbers by the pharmacy of the University Hospital Marburg without revealing the assignment of BoNT or placebo. The pharmacy received an identification list from the Coordination Center for Clinical Trials, which allocated 4-digit coding numbers to the four treatment groups. After randomization of a patient, the pharmacist was informed about the assigned 4-digit coding number by the Coordination Center for Clinical Trials and labelled the injections with the particular side (left and right) according to the identification list. The assignment was neither known to the treating physician nor to the patient nor to the evaluator. The assignment was only known to the Coordination Center for Clinical Trials and to the pharmacy.

### Safety and tolerability

In accordance with the national law, a pharmacovigilance system was established to record and assess any adverse events (AEs) related to BoNT application as study medication. Serious AEs were reported to the sponsor immediately. In case of suspected unexpected serious adverse reactions (SUSARs), these cases were reported to the respective authorities according to applicable law. In case of severe AEs, the investigator decided case by case whether to terminate the individual treatment or not. The incidence and severity of all AEs were graded according to the EMA's note for guidance on clinical safety data management, ICH E2A.

Changes in vital signs, routine laboratory parameters, physical examination findings, electrocardiography, and medical conditions during and after radiochemotherapy were assessed throughout the study with a minimum follow-up of six months (6–13 months, mean 8.3 months). Patients were monitored for radiochemotherapy related toxicities according to NCI common toxicity criteria (CTC). AEs were recorded since randomization until 30 days after the last regular study visit of each patient. Patients with AEs were treated accordingly and ongoing AEs at the end of the individual study treatment were followed and treated until resolution. The stopping criteria comprised occurrences of AEs and serious AEs that did not allow the continuation of the treatment according to the study protocol. In addition, the principal investigator could discontinue the study for medical reasons in a patient’s interest and also in cases of non-compliance of the patient. A patient’s own volition to discontinue the study and pregnancy were other stopping criteria.

### Quantitative scintigraphic studies

In addition to safety and tolerability, another primary outcome measure of the study was defined as the difference between the scintigraphically assessed radionuclide uptakes of the BoNT/A or BoNT/A and B treated gland and the placebo (NaCl) treated contralateral gland for each patient (side difference) before and after BoNT injection and radiochemotherapy (pre-post side difference). The secondary outcome was the salivary excretion fraction (SEF), which was defined as the ratio between excreted radioactivity triggered by drinking lemon juice and the total amount of radioactivity within the gland before drinking.

Taking the uptake difference between the two glands for each patient reduces the within-patient variability due to situation-dependent parasympathetic stimulation, which affects both glands in the same way (e.g., just thinking of lemon juice could cause salivation before drinking it). Because gland functionality may already differ between patients at baseline, the evaluation of the pre-post side difference instead of the side difference after radiation therapy alone reduces the between-patient variability of the treatment effect. The information contributed by the patients can hence be exploited more efficiently.

After injection of 74 MBq ^99m^ Tc-pertechnetate, scans were acquired during 45 min using a digital gamma camera (Symbia, Siemens, Erlangen, Germany, lateral resolution: 128x128 pixels) equipped with a Low Energy High Resolution (LEHR) collimator. Raw data were processed using the software Syngo (Siemens, Erlangen, Germany). Uptake of the salivary glands was determined by the region of interest (ROI) technique and was calculated as percentage of the total amount of administered radionuclide that was taken up by the respective salivary gland. All ROIs and the submandibular gland region were defined manually for each patient individually. In order to account for the background activity, which was measured in the supraclavicular area, the difference between the accumulation in the gland ROI and the accumulation in the background ROI was used. For evaluation of SEF, 4 ml of lemon juice were administered orally 15 min after injection of ^99m^ Tc-pertechnetate. Scintigraphic studies, i.e., assessment of uptake and SEF, were performed before radiotherapy (baseline) and 4–6 months after the end of the radiotherapy (follow-up). It has been shown that patients’ salivary function declines significantly 3–4 months after the end of radiotherapy and the late phase of radiation damage starts after 4 months [12].

### Statistical analysis of scintigraphic data

Differences between outcome and baseline values are referred to as change scores, the analysis of which is defined as “simple analysis of change scores (SACS)” [13]. The uptake and SEF pre-post side differences are such change scores and were hence analyzed using SACS.

As each patient served as his/her own control, the data were matched and, hence, analyzed using the Wilcoxon signed rank test. The alternative two-sided hypothesis for each of the two treatment groups (BoNT/A as well as BoNT/A-B) corresponds to a difference in primary outcome measure, i.e., the uptake pre-post side difference, between treated and untreated glands different from 0. An expected value of 0 corresponds to no treatment effect of BoNT, i.e., the null hypothesis, which is equivalent to no difference in treatment effects between BoNT and placebo. P-values ≤ 0.05 were considered nominally significant. Power calculations assumed a mean treatment effect between the verum-treated and placebo-treated glands of 2 standard deviation (SD) units as had been observed in studies with rats [9,10]. Hence, assuming a mean treatment effect of 2 SD units, normally distributed data, a sample size of 6 patients, and a type I error rate of 5%, the Wilcoxon signed rank test has a power of 87%. As a result, 6 patients were recruited for each dose group, leading to a combined sample size of 12 patients for both treatments. The sample size of the present study was large enough to also allow for the side-adjusted testing for differences in treatment effects between BoNT/A vs. BoNT/A-B by the van-Elteren test [14]. The two-sided null hypothesis of each stratum (right verum-treated and left verum-treated) corresponds to: values of both treatment groups are drawn from the same distribution, i.e., treatment effects are the same for BoNT/A and BoNT/A-B. The combined alternative hypothesis corresponds to a difference in treatment effects across the strata.

All analyses for the uptake difference, as described above, were repeated for the SEF difference.

### Additional, non-confirmatory statistical analyses

Generally, an analysis of covariance (ANCOVA) that includes baseline values as a covariate is the preferred approach to maintain precision of the treatment effect estimate as well as to control type I error and power of the test [15]. However, this method is not applicable in case of small sample sizes, such as the treatment groups with only 6 patients in the current study. ANCOVA can also be used to assess the strength of correlation between baseline and outcome values in terms of the Pearson correlation coefficient ρ estimated by r. This is important because the power of SACS crucially depends on a sufficiently high positive correlation between baseline and outcome values. Accordingly, further analyses were run (i) to assess the strength of correlation between baseline and outcome values in terms of the correlation coefficient r, (ii) to test for treatment effects within each treatment group based on unadjusted outcome values, i.e., without subtracting baseline values, and (iii) to test for treatment differences between the two treatment groups based on unadjusted outcome values while taking a potential effect of the side (right/left) of verum and placebo application into account. Specifically, we used (i) ANCOVA with baseline values as the only covariate in the model, (ii) the Wilcoxon signed rank test on unadjusted outcomes in each treatment group, and (iii) the van-Elteren test to compare unadjusted outcome values between the BoNT/A and BoNT/A-B treatment groups. The test results of the additional analyses are not interpreted in a confirmatory manner but are meant to explore the data regarding baseline-outcome correlations and differences in the unadjusted outcome values.

All analyses were carried out using the package *coin* [16,17] of the statistical software *R* [18] to calculate Wilcoxon signed rank and van-Elteren tests, which yield exact p-values.

## Results

### Patient characteristics

**Fig 1:**
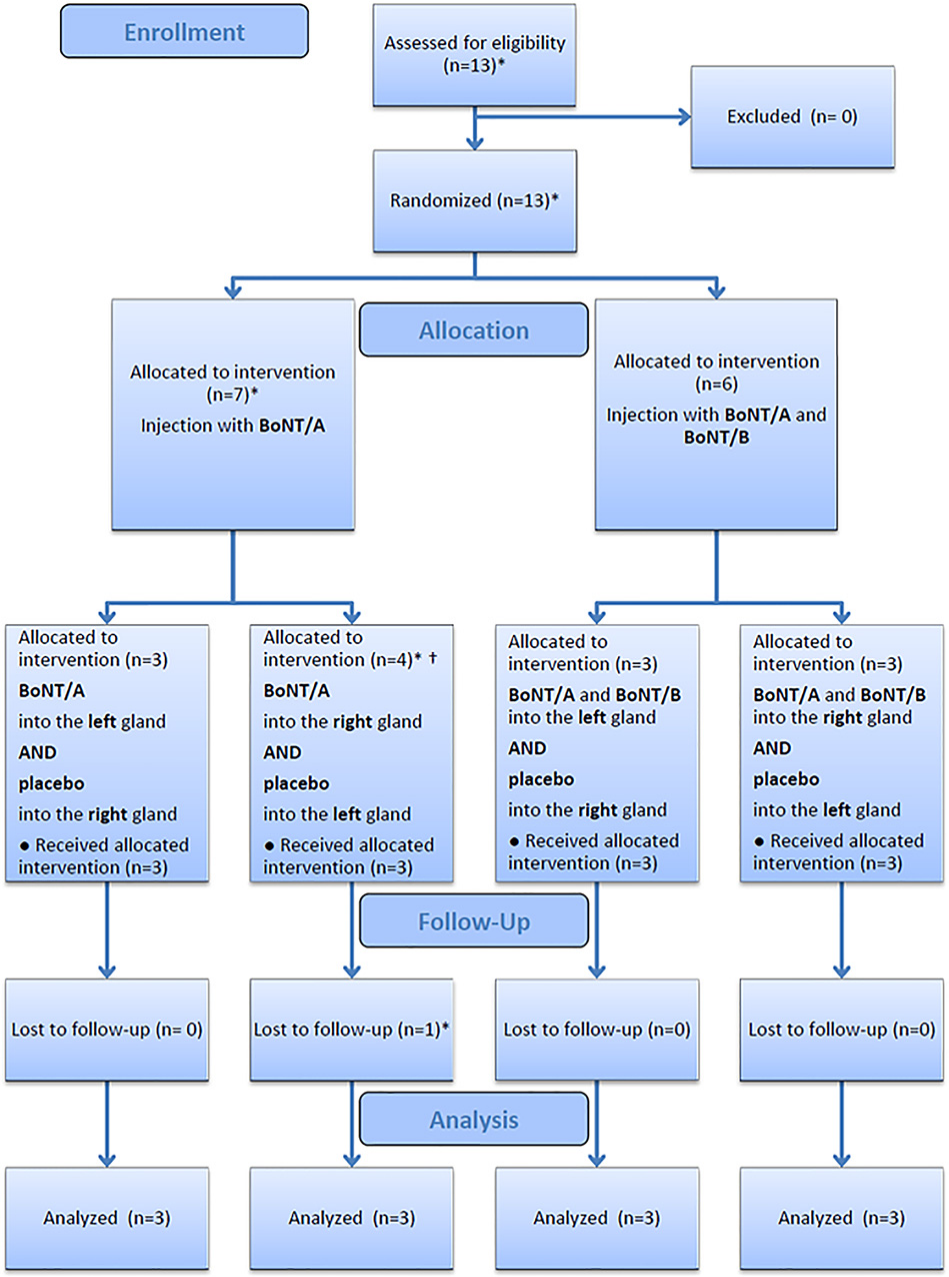
Overview of the participant flow. * The study was originally designed for 12 patients with balanced randomization of 3 patients to each of the 4 study arms. One patient in treatment group BoNT/A (right gland) was not treated according to the study protocol and therefore excluded from the study. An additional patient was recruited and assigned to the treatment group of the excluded patient to retain the balanced allocation. The assignment was only known to the Coordination Center for Clinical Trials and double-blindness was maintained. The original patient refused radiochemotherapy and did not undergo post-treatment scintigraphy and therefore was excluded from the analysis. † One patient originally allocated to group BoNT/A (right gland) was actually treated with BoNT/A in the left gland and placebo in the right gland due to mix-up of the injections designated for the two sides. According to actual treatment, this patient was assigned to treatment group BoNT/A (left gland) and a new patient was allocated to group BoNT/A (right gland) to retain the balance. The actual group assignment was only known to the Coordination Center for Clinical Trials and double-blindness was maintained. Because of the double-blinded nature of the trial, it can be assumed that the treatment error occurred accidentally and therefore did not lead to any selection bias.

Twelve patients (2 females, 10 males; mean age at diagnosis: 55.4 years, range: 45–66 years) with stage III and IV HNSCC were included in the analysis of the study (see Fig 1 for a chart of the participant flow). The clinical data, cancer stages, time interval between BoNT injection and start of radiochemotherapy, and details about the radiochemotherapy are summarized in Table 1. One patient (no. 11) could not meet his original appointment for the beginning of radiochemotherapy and was hence injected with BoNT 6 weeks prior to treatment as compared to 2–3 weeks in all other cases.

**Table 1:** Cancer characteristics and applied radiochemotherapy in patients of the present study with HNSCC (n = 12+1, see also Fig 1). 5FU = 5-Fluoruracil, Cis = Cisplatin, Mito = Mitomycin C. Treatment groups: 1: BoNT/A injected in the right gland, placebo in the left gland; 2: BoNT/A in the left gland, placebo in the right gland; 3: BoNT/A and B in the right gland, placebo in the left gland; 4: BoNT/A and B in the left gland, placebo in the right gland.

Patient number	Treatment group	Age(y)	Sex	Localization of the primary tumor site	Grading	Clinical stage	Time period between BoNT injection and starting radiotherapy (d)	Total absorbed radiotherapy dose (Gy)	Chemotherapy
1	2	56	m	hypopharynx	2	T4N2cM0	13	64.6	5FU, Cis 3 cycles
2	2	66	m	oropharynx	2	T4N2cM0	14	70	no chemotherapy
3	2	61	f	supraglottic	2	T3N1M0	21	72 *	5FU, Cis 3 cycles
4	1	51	m	oropharynx	2	T4N2cM0	14	71	5FU, Mito 1 cycle
5	4	49	m	oropharynx	2	T3N2cM0	20	72	5FU, Cis 6 cycles
6	3	49	m	hypopharynx	2	T2N2cM0	16	72	5FU, Cis 3 cycles
7	3	58	m	oropharynx	2	T3N2bM0	19	72.6	5FU, Mito 2 cycles
8	4	59	m	oropharynx	2	T2N1M0	14	69.4	5FU, Cis 6 cycles
9	1	59	m	oropharynx	2	T3N0M0	14	70.6	5FU, Cis 6 cycles
10	4	45	f	oral cavity	2	T4N2bM0	13	72 *	5FU, Cis 3 cycles
11	3	60	m	hypopharynx	3	T2N2cM0	44	72	5FU, Cis 4 cycles
12	1	52	m	oropharynx	2	T2N2cM0	14	72	5FU, Cis 6 cycles
13	1	61	m	oropharynx	2	T3N2cM0	NA	NA	NA

* no hyperfractionated radiotherapy

**Fig 2:**
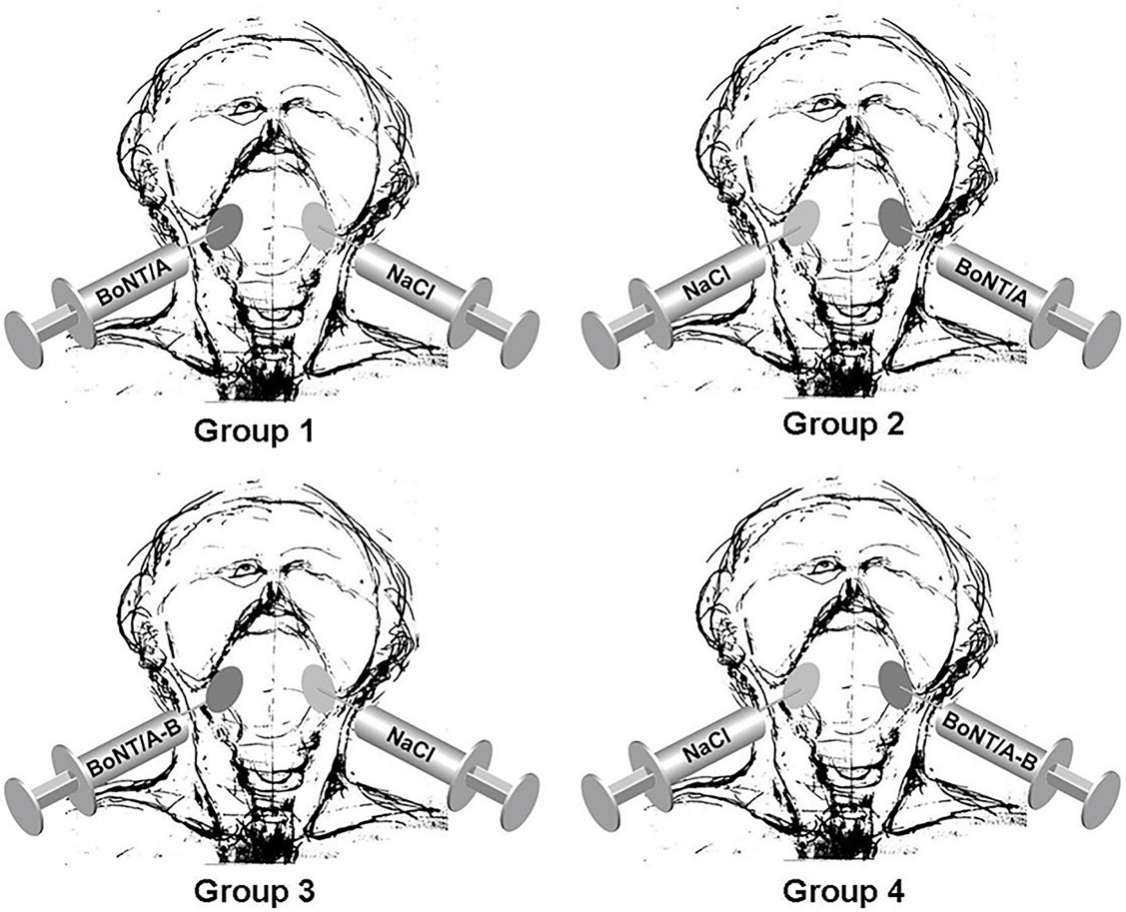
Graphical depiction of the allocation process leading to the four treatment groups.

Fig 2 illustrates the allocation of patients to treatment groups. Radiotherapy was completed in all patients. The dosimetric data of the submandibular glands on both sides are summarized in Table 2, which shows comparable radiation doses. There were some variations in the chemotherapy protocol mostly because of infections, leukopenia, and renal insufficiency in some patients (see Table 1).

**Table 2:** Dosimetric parameters of the left and right submandibular glands for each patient in Gray. D_min_ = minimum dose, D_max_ = maximum dose, D_mean_ = mean dose, SD = standard deviation. For an explanation of treatment group numbers see legend of Table 1.

		Left submandibular gland			Right submandibular gland		
Patient number	Treatment group	D_min_	D_max_	D_mean_	D_min_	D_max_	D_mean_
1	2	53.7	67.5	63.4	53.2	52.2	62.6
2	2	62.7	69.1	67.4	56.5	68.4	66.4
3	2	61.7	76.4	72.9	65.7	75.3	72.6
4	1	58.3	69.3	65.4	58.6	70.0	66.7
5	4	59.0	77.5	68.4	59.2	76.3	68.0
6	3	57.5	77.0	67.5	68.0	77.8	74.0
7	3	65.4	75.3	71.2	67.7	77.1	72.8
8	4	59.4	74.1	69.7	60.4	76.7	69.8
9	1	70.7	77.6	74.2	70.6	77.6	73.9
10	4	70.2	75.8	72.3	70.1	75.6	60.4
11	3	57.3	78.7	74.0	55.2	77.5	73.2
12	1	56.2	77.0	69.8	67.1	77.7	73.8
mean (SD)		60.2 (6.8)	74.6 (3.8)	69.7 (3.4)	62.7 (6.2)	73.5 (7.4)	69.5 (4.7)

### Safety and tolerability

The medication with BoNT was generally well tolerated. The reported AEs and serious AEs were likely unrelated to study medication. Three serious AEs were reported in one patient six months after BoNT injection. This patient showed mild symptoms of dehydration. Further AEs of this patient included mild to moderate temporary symptoms of nervous system and psychiatric disorders. The patient was treated conservatively for few days in the hospital. One other patient developed distant metastases seven months after the first diagnosis and BoNT injection as a serious AE. Taken together, AEs were documented in ten patients. The most frequently reported AEs were: leukopenia in 4, symptoms of dehydration, general physical health deterioration, radiation skin injury in 2, and two times bleeding from the tracheostoma after changing of tracheal cannula in 1 patient. All AEs, except the above-mentioned metastases, were classified as mild or moderate. An overview of the AEs is given in Table 3.

**Table 3:** Overview of adverse events.

Patient number	Treatment group	MedDRA PPT	MedDRA SOC	Serious	Intensity/grade
5	4	Leukopenia	Blood and lymphatic system disorders	no	mild/1
5	4	Partial seizures	Nervous system disorders	no	mild/1
5	4	Hyponatraemia	Metabolism and nutrition disorders	no	mild/1
9	1	Renal failure	Renal and urinary disorders	no	moderate/2
9	1	Atrial fibrillation	Cardiac disorders	no	moderate/2
11	3	Post procedural haemorrhage	Injury, poisoning and procedural complications	no	mild/1
11	3	Post procedural haemorrhage	Injury, poisoning and procedural complications	no	mild/1
11	3	General physical health deterioration	General disorders and administration site conditions	no	mild/1
2	2	Stomatitis	Gastrointestinal disorders	no	mild/1
2	2	Radiation skin injury	Injury, poisoning and procedural complications	no	mild/1
2	2	Metastasis	Neoplasms benigns, malignant and unspecified (incl. cysts and polyps)	yes	severe/3
10	4	Pneumonia	Infections and infestations	no	moderate/2
10	4	Anaemia	Blood and lymphatic system disorders	no	mild/1
7	3	Radiation skin injury	Injury, poisoning and procedural complications	no	mild/1
8	4	Leukopenia	Blood and lymphatic system disorders	no	mild/1
8	4	Hypokalaemia	Metabolisms and nutrition disorders	no	mild/1
4	1	Dehydration	Metabolism and nutrition disorders	no	mild/1
4	1	Leukopenia	Blood and lymphatic system disorders	no	mild/1
1	2	Leukopenia	Blood and lymphatic system disorders	no	mild/1
1	2	Seizure	Nervous system disorders	no	mild/1
1	2	Dehydration	Metabolism and nutrition disorders	no	mild/1
1	2	Cerebral ischaemia	Nervous system disorders	yes	moderate/2
1	2	Dehydration	Metabolism and nutrition disorders	yes	mild/1
1	2	Apathy	Psychiatric disorders	yes	moderate/2
12	1	General physical health deterioration	General disorders and administration site conditions	no	mild/1

Radiochemotherapy was generally well tolerated. Toxicity of radiochemotherapy including xerostomia and mucositis was dose adequate. None of the patients experienced side effects including mouth dryness, dysphagia, dermatitis, mucositis, sense of smell, and nausea of more than grade 2 according to common toxicity criteria (CTC) in the follow-up time after radiochemotherapy.

### Analysis of scintigraphic data

A significant difference in submandibular salivary gland function after radiotherapy in terms of ^99m^ Tc-pertechnetate uptake was not observed between glands injected with either BoNT or placebo (pBoNT/A = 0.84; pBoNT/A-B = 0.56). The results of the Wilcoxon signed rank tests for the uptake differences are presented in Table 4. The Hodges-Lehmann point estimate (H-L PE) of the treatment effect was slightly negative in the BoNT/A (H-L PEBoNT/A = -4.07e-05) and slightly positive in the BoNT/A-B treated glands (H-L PEBoNT/A-B = 7.62e-05). We also found no significant difference in treatment between BoNT and placebo in terms of SEF (pBoNT/A = 0.44; pBoNT/A-B = 0.44) (Table 5). The H-L PE of the SEF treatment effects of both treatment groups were positive (H-L PEBoNT/A = 4.68e-02; H-L PEBoNT/A-B = 8.10e-02). The results of the van-Elteren tests for comparison of treatment effects between BoNT/A vs. BoNT/A-B were neither significant regarding the uptake differences (pUptake = 0.68) nor the SEF differences (pSEF = 0.78). Raw scintigraphic data were presented in S3 Table and S6 Text.

**Table 4:** Results of the Wilcoxon signed rank test for the baseline adjusted uptake difference. n: number of patients in each dose group.

Dose group (n)	Two-sidedp-value	Hodges-Lehmann point estimate(H-L PE)^§^	95% confidence interval for H-L PE
BoNT/A (6)	0.84	-4.07e-05	-9.31e-04; 4.97e-04
BoNT/A-B (6)	0.56	7.62e-05	-3.72e-04; 8.22e-04

^§^ The H-L PE is the effect estimate of the Wilcoxon signed rank test, which evaluates the median of all pair-wise averaged differences in outcome. It is also known as the pseudomedian. A pseudomedian of 0 corresponds to the null hypothesis of no differences in outcome between placebo- and verum-treated glands.

**Table 5:** Results of the Wilcoxon signed rank test for the baseline adjusted SEF difference.

Dose group (n)	Two-sidedp-value	Hodges-Lehmann point estimate(H-L PE)^§^	95% confidence interval for H-L PE
BoNT/A (6)	0.44	4.68e-02	-0.17; 0.24
BoNT/A-B (6)	0.44	8.10e-02	-8.42e-02; 0.57

^§^ See annotation in Table 4.

### Additional, non-confirmatory analyses

**Fig 3:**
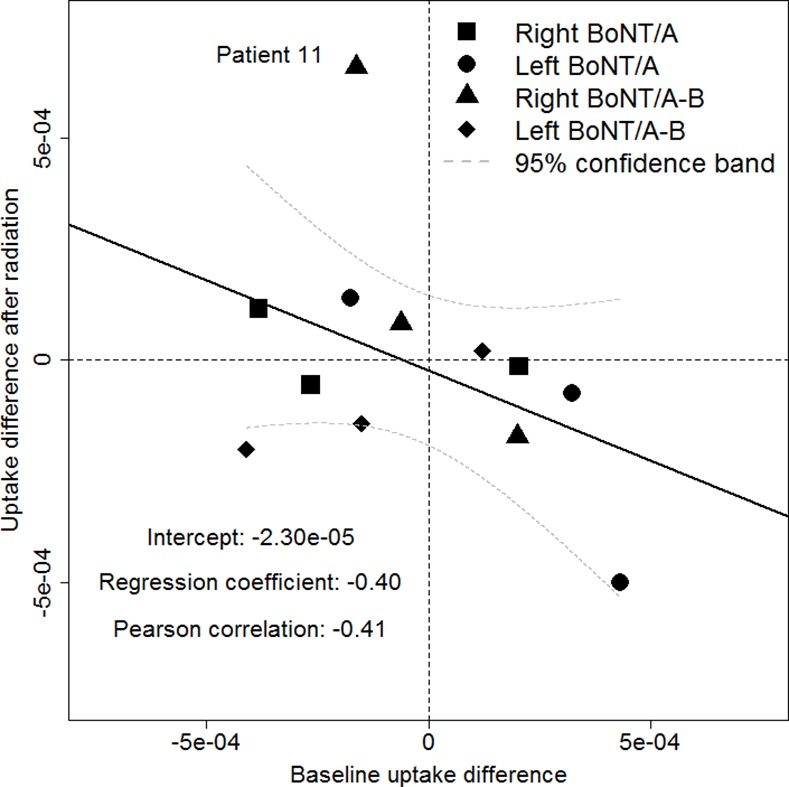
Results of the ANCOVA analyses for the uptake difference of the submandibular glands after radiotherapy.

**Fig 4:**
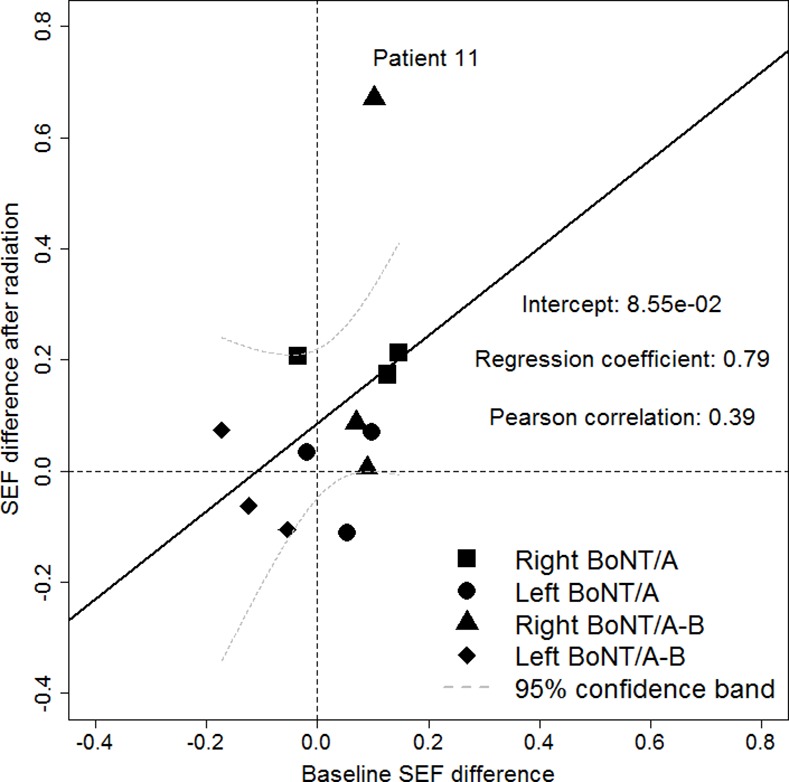
Results of the ANCOVA analyses for the SEF difference of the submandibular glands after radiotherapy.

**Fig 5:**
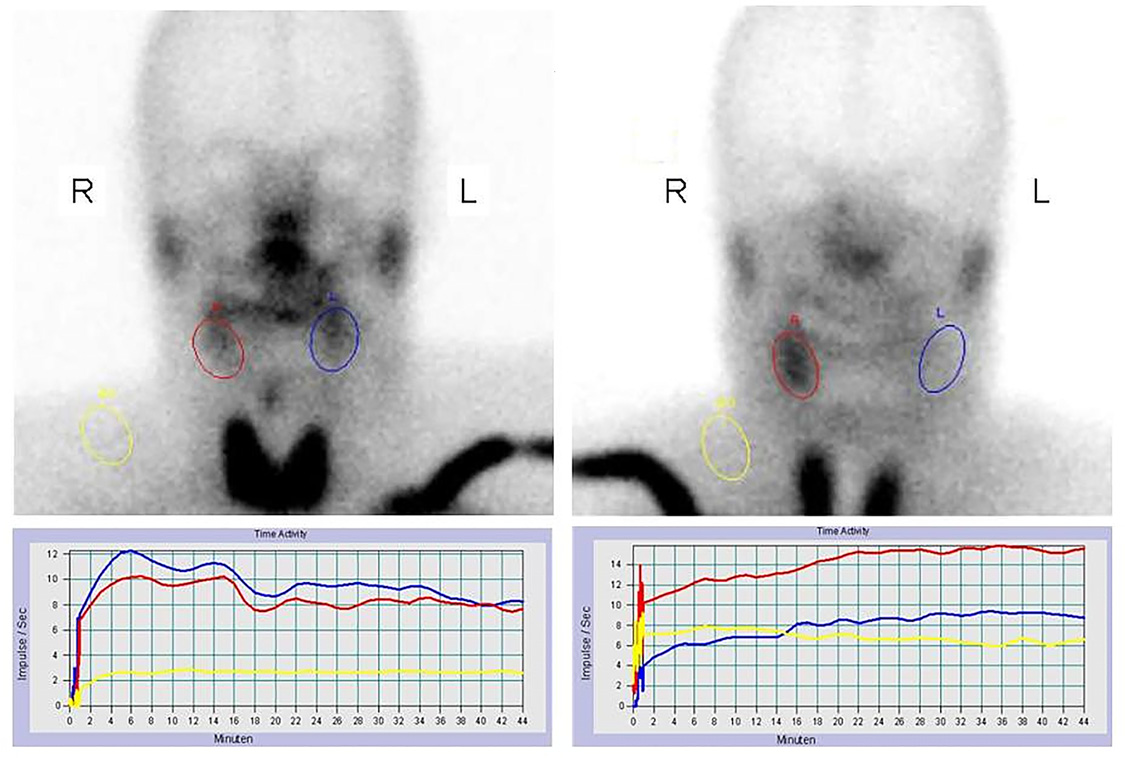
Salivary gland scintigraphy before (left) and after (right) radiotherapy of patient number 11. A consistently higher uptake level of the right submandibular gland area treated with BoNT compared to the control gland on the opposite side was demonstrated. Time activity curves on quantitative scintigraphy of the submandibular gland before (left) and after (right) radiotherapy are shown. Red: right gland, blue: left gland, yellow: background activity.

The results of the ANCOVA analyses are given in Fig 3 for the uptake difference and in Fig 4 for the SEF difference. The correlation between baseline and outcome values was -0.41 for the uptake difference. The intercept of the model (-2.30e-05) corresponds to the baseline-adjusted joint treatment effect of BoNT/A and BoNT/A-B. The correlation between baseline and outcome SEF differences was 0.39 with a baseline-adjusted joint treatment effect of BoNT/A and BoNT/A-B of 8.55e-02. Obviously, the regression lines for both the uptake and the SEF difference are highly influenced by a single patient labeled number 11. This demonstrates the difficulty to accurately estimate the correlation given the small sample size. Here, patient 11, who belongs to the BoNT/A-B dose group, showed a remarkable treatment effect, which was also confirmed by visual analysis of scintigraphic pictures (Fig 5). The patient´s uptake difference was 8.22e-04 and the SEF difference was 0.57. It is of note that the placebo-treated gland of patient 11 was almost completely destroyed after radiotherapy, whereas the verum-treated gland still showed normal functionality. The Wilcoxon signed rank tests on unadjusted outcomes in each treatment group did not show any significant results (see Table 6 for the uptake differences and Table 7 for the SEF differences). In the same line, the van-Elteren tests to compare unadjusted outcome values between the BoNT/A and BoNT/A-B treatment groups for the uptake and the SEF differences were also not significant (pUptake = 0.915 and pSEF = 0.605).

**Table 6:** Results of the Wilcoxon signed rank test for the unadjusted uptake difference.

Dose group (n)	Two-sided p-value	Hodges-Lehmann point estimate(H-L PE)^§^	95% confidence interval for H-L PE
BoNT/A (6)	0.84	-3.52e-05	-4.99e-04; 1.40e-04
BoNT/A-B (6)	0.84	-4.48e-05	-2.00e-04; 6.59e-04

^§^ See annotation in Table 4.

**Table 7:** Results of the Wilcoxon signed rank test for the unadjusted SEF difference.

Dose group (n)	Two-sided p-value	Hodges-Lehmann point estimate(H-L PE)^§^	95% confidence interval for H-L PE
BoNT/A (6)	0.16	0.12	-0.11; 0.21
BoNT/A-B (6)	0.56	3.97e-02	-0.11; 0.67

^§^ See annotation in Table 4.

## Discussion

Preservation of the submandibular gland function is a crucial factor in preventing serious side effects of radiotherapy to the head and neck region as these glands produce the greatest saliva volume in the non-stimulated state. The mucin-rich saliva of the submandibular gland serves as mucosal lubricant that mainly contributes to the patient’s subjective sense of moisture [19].

Depending on tumor location and location of involved nodal metastases, modern high precision radiotherapy techniques such as intensity modulated (IMRT) and image guided techniques enable substantial sparing of normal tissues, e.g. (parts of the) parotid glands, in many cases. On the contrary, due to the close proximity of the submandibular glands to the jugulodigastric nodes, which are the first echelon nodes in most head and neck cancers [20], the submandibular glands can rarely be spared from the irradiation but they receive a relevant or even the prescribed irradiation dose. Based on this background, our previous experiments about radioprotective agents and the present study focus on the submandibular glands.

Application of BoNT as an established agent for different diseases has been shown to be effective in the treatment of hypersalivation [21]. Intraglandular injection of BoNT causes inhibition of acetylcholine release at the neuroglandular junction and leads to a transitory pharmacological denervation of the salivary glands associated with reduced salivary secretion [22]. We recently analyzed the effect of locally injected BoNT in salivary glands of the rat at the cellular and ultrastructural level. We found that intraglandular application of BoNT induces morphological and functional changes, indicated by temporary glandular atrophy with reduced amounts of secretory granules of the acinar cells [10]. These effects may be due to glandular denervation induced by the inhibition of the SNAREs (soluble N-ethylmaleimide-sensitive fusion protein attachment protein receptors) involved in the acetylcholine release at the neuroglandular junction, especially by inhibition of those involved in exocytosis of the granula of the acinar cells.

Temporary acinar atrophy in conjunction with chemical denervation of the salivary glands could be of interest for prevention of radiation-induced sialadenitis. The decisive pathogenic factor in irradiation-induced sialadenitis is a direct radiation effect on the glandular tissue. The radiation dose and the dose distribution are directly related to the severity of the glandular damage [23]. The quantity of secretory granules of the acinar cells also counts as an important pathogenic factor [24,25]. Therefore, intraglandular application of BoNT prior to radiation could lead to glandular atrophy with reduction of acinar granules during the time of irradiation, which might significantly reduce the radiation sensitivity of the glandular acini.

For in vivo verification of the mentioned hypothesis of preservation of acinar cells during radiotherapy, we previously established a rat model for functional studies on salivary glands. Scintigraphy with ^99m^ Tc-pertechnetate was shown to be appropriate for functional studies of salivary glands of rats [26]. Using this animal model of radiation-induced sialadenitis, we have shown by salivary scintigraphic measurements and histomorphological studies that the intraglandular injection of BoNT prior to irradiation significantly reduces functional and structural radiation-induced damage of salivary glands [9].

Based on this previous work, the present study provided the first clinical experience of the safety profile and the potential function preserving effect of BoNT after irradiation of submandibular glands in humans. This study revealed that BoNT can be safely combined with radiochemotherapy. We did not set any prior condition regarding the maximal rate of toxicity of BoNT/A and B as a criterion for good safety. However, since none of the observed adverse events are likely to be due to the study medication, it is warranted to proceed to a phase II clinical trial as the next step.

Analysis of the treatment groups did not show a significant difference in the salivary function after injection of BoNT or placebo in terms of uptake or SEF difference. Only in one patient we observed a substantial uptake and SEF difference. This patient was treated with a combination of BoNT/A and B. The previously mentioned animal study revealed that most significant morphological changes occurred in glands treated with both BoNT/A and B [10]. BoNT/A cleaves SNAP-23(25) (synaptosomal-associated protein) and BoNT/B VAMP (vesicle-associated membrane protein, synaptobrevin), enzymes involved in the release of acetylcholine at the presynaptic membrane of parasympathetic nerves. In this way, a temporary chemical denervation of the target organ is established. The cleavage of different SNAREs in the presynaptic and acinar cell membrane might be responsible for the more pronounced effects of the combination of BoNT/A and B.

Comparison of the evaluated data of this patient with others revealed that the time period between BoNT injection and start of radiotherapy was 6 weeks in that patient compared to 2–3 weeks in all other cases, which might be the reason for the appreciable effect in that case. Other clinical and treatment differences or study protocol violations than those described in this paper (see also Fig 1) did not exist.

Admittedly, the trial was limited by the small number of patients under study. However, in consideration of the above-mentioned findings in rats, we expected a comparable large effect in humans and hence did not intend to enroll more patients in this phase I clinical trial than required. Prior to this trial, sample size calculations were performed, based on the effect size found in previous work with rats [9,10]. To exploit the data on the small number of patients more efficiently, we used an elaborate study design in which each patient served as its own control in order to reduce variability in outcome measures within and between patients. This study design, which is similar to the classical cross-over design, proved to be appropriate in our study. Yet, our findings neither showed a significant effect with regard to the uptake nor the SEF difference. This might be due to the reduced precision of the scintigraphic measurements in humans as compared to the measurements in rats. In addition, the applied simple analysis of change scores (SACS), i.e., subtracting the baseline values from those measured after treatment, may not always be the best option. This is due to the fact that SACS is only superior in terms of power to an analysis of raw outcome values if the correlation between baseline and outcome values is larger than 0.5 (see Figs 3 and 4). An ANCOVA, as shown in Figs 3 and 4 for the joint treatment effect of BoNT/A and BoNT/A-B, could be applied instead to overcome this problem. In addition, it is conceivable that findings and effect sizes from studies with rats might not be completely transferable to humans, which calls for further investigation of the mode of action of BoNT/A and BoNT/B, respectively.

Our study provided the first clinical experience of the effect of BoNT injected in salivary glands prior to combined radiochemotherapy in patients with HNSCC. While the study proved excellent tolerability, the effect on organ function preservation could not be demonstrated with our sample of 12 patients. There are hints pointing at an improved efficacy with a modified timing and possibly dosing schedule of BoNT/A and B, which should be further evaluated in future studies.

### Competing interests

This study was financially supported by the Von-Behring-Röntgen Foundation, Marburg, Germany, Grant No. 57–0011. Furthermore, this work was supported within the Munich Center of Health Sciences (MC-Health), Ludwig-Maximilians-Universität, as part of LMUinnovativ. All authors have declared no conflicts of interest.

All authors have completed the Unified Competing Interest form at http://www.icmje.org/coi_disclosure.pdf and declare: no financial relationships with any organisations that might have an interest in the submitted work in the previous 3 years; no other relationships or activities that could appear to have influenced the submitted work.

## Supporting Information

S1 Table Study synopsis. (DOCX)

S2 Table Consort checklist. (DOC)

S3 Table Raw scintigraphic data. (CSV)

S1 Text Study protocol. (PDF)

S2 Text Study protocol, additions 1. (PDF)

S3 Text Study protocol, additions 2. (PDF)

S4 Text Sample case report form. (PDF)

S5 Text ICMJE Form for Disclosure of Potential Conflicts of Interest. (PDF)

S6 Text Caption to S3 Table. (PDF)
