# Supplementary material for: Safety and Efficacy of Botulinum Toxin to Preserve Gland Function after Radiotherapy in Patients with Head and Neck Cancer: A Prospective, Randomized, Placebo-Controlled, Double-Blinded Phase I Clinical Trial
Source: PLoS One. 2016 Mar 18;11(3):e0151316. doi: 10.1371/journal.pone.0151316 (PMC4798778; doi:10.1371/journal.pone.0151316)
Supplement: S1 Table — (DOCX) [file pone.0151316.s001.docx]

Study Synopsis

| **Study title** | The value of intraglandular application of botulinum toxin for prevention of radiation-induced sialadenitis in head and neck cancer |
| --- | --- |
| **Sponsor** | Philipp University Marburg |
| **Coordinating Investigator (CI)** | Prof. Dr. med. Afshin Teymoortash  Head of the Department of [Otolaryngolog](http://www.dict.cc/englisch-deutsch/otolaryngologist.html)y, head and neck Surgery, Philipp University of Marburg, Baldingerstrasse  35043 Marburg, Germany |
| **Studycenter** | 1 Center, Germany |
| **Study design / Study phase and** | Four-armed, monocenter, randomized, double-blind phase I-study |
| **Indication** | squamous cell carcinoma of the head and/or neck |
| **Study medication (IMP)** | - BoNT/A (botulinum toxin A) and BoNT/B (botulinum toxin B) - Placebo (NaCl) |
| **Study arms** | - 3 patients: direct application of BoNT/A (15U) into submandibular gland right, and NaCl into submandibular gland left; single application before radiochemotherapy. - 3 patients: direct application of BoNT/A (15U) into submandibular gland right, and NaCl into submandibular gland left; single application before radiochemotherapy. - 3 patients direct application of BoNT/A (15U) and BoNT/B (750U), into submandibular gland right, and NaCl into submandibular gland left; single application before radiochemotherapy. - 3 patients: direct application of BoNT/A (15U) and BoNT/B (750U), into submandibular gland left, and NaCl to the submandibular gland right; single application before radiochemotherapy. |
| **Study design** | In patients with a squamous cell carcinoma of the head and neck, primary conventional chemoradiotherapy of the neck shall be performed. The submandibular glands of both sides are located within the radiation field. In each patient BoNT (botulinum toxin) shall be once injected into the right or left submandibular gland before radiochemotherapy, depending on the result of the randomization. The same volume of NaCl will be applicated to the mutual submandibular gland, which serves as a control gland. Functional testing of both submandibular glands will be performed before and at least 4 months after completion of the radiochemotherapy by scintigraphic methods. |
| **Planned number of patients** | 12 patients |
| **Inclusion criteria** | - Patients with a squamous cell carcinoma of the head and neck - Signed informed consent form - Cognitive and physical ability to understand the aim of the study and to adhere to the protocol - Age ≥ 40 years - Gender: female and male patients - Fertile woman over 40 years have to use one of the following contraceptive methods: contraceptive pill and contraceptive coil, since at least to one month before study start, barrier with spermicide (condom or diaphragm), tube ligature. Fertile woman are defined as women whose last menstruation dates back no longer than one year - No functional restrictions and sides differences detected by the initial scintigraphy of the submandibular glands |
| **Exclusion criteria** | - Condition after [extirpation](http://www.dict.cc/englisch-deutsch/extirpation.html) of the submandibular glands - Known illnesses of the submandibular glands - Concomitant medication, which affect the submandibular glands - Condition after previous radiotherapy of cervical soft tissue - Pregnancy - Lactation - Irenat-therapy, - Hypersensitivity to Botulinum toxin or one of the other components |
| **Endpoints** | **Primary endpoint**  Proportional uptake of radionuclide after treatment with BoNT or placebo. The primary endpoint will be determined via scintigraphy before and after study treatment.  **Secondary endpoint**  Difference of the ejection fraction of the submandibular glands either treated with BoNT or placebo after administration of an irritant drink |
| **Statistical methods** | Wilcoxon signed rank Test, Mann-Whitney-U-Test, van-Elteren test, and descriptive analysis |
| **Study duration** | Approx. 7-8 months per patient  Approx. 23 months all together |
