# Supplementary material for: Safety and Efficacy of Botulinum Toxin to Preserve Gland Function after Radiotherapy in Patients with Head and Neck Cancer: A Prospective, Randomized, Placebo-Controlled, Double-Blinded Phase I Clinical Trial
Source: PLoS One. 2016 Mar 18;11(3):e0151316. doi: 10.1371/journal.pone.0151316 (PMC4798778; doi:10.1371/journal.pone.0151316)
Supplement: S1 Text — (PDF) [file pone.0151316.s004.pdf]

Studienprotokoll

Funktionsszintigraphische Untersuchungen zur Überprüfung der radioprotektiven Wirksamkeit intraglandulär applizierten Botulinum-Toxins (BoNT) auf die Gl. submandibularis bei Patienten mit einem Plattenepithelkarzinom der oberen Luft- und Speisewege

Autoren:

Prof. Dr. med. Afshin Teymoortash, S. Harnisch, Prof. Dr. rer. nat. Konstantin Strauch

Studienprotokollnr: EudraCt. Nr.: 2009-014576-22

Protocol-Code-Nr.: KKS-122

Leiter Klinische Prüfung: Prof. Dr. med. Afshin Teymoortash

Sponsor Philipps-Universität
Biegenstrasse 10
35037 Marburg

Datum/Version Prüfplan V01 F–2009.07.23

Dieser Prüfplan unterliegt einschließlich aller seiner Teile dem Urheberrecht. Er ist Eigentum des Studienleiters Prof. Dr. A. Teymoortash, des Biometrikers Prof. Dr. K. Strauch und des Koordinierungszentrums für Klinische Studien (KKS) der Philipps-Universität Marburg. Er wird den Studienteilnehmern vertraulich zur Verfügung gestellt. Dieser Prüfplan darf nicht ohne schriftliche Genehmigung des Studienleiters und des KKS an Dritte weitergegeben werden, vervielfältigt, eingespeichert oder in elektronischen Systemen weiterverarbeitet werden.

Prüfplanunterschriften

Ich habe dieses Protokoll gelesen und stimme zu, die Studie in Übereinstimmung mit allen Festlegungen dieses Protokolls sowie ICH-GCP, der Deklaration von Helsinki (Version 1996) und des AMG und der GCP-Verordnung durchzuführen.
Außerdem stimme ich zu, sämtliche Informationen bezüglich dieser Studie geheim zu halten

Bevollmächtigter des Sponsors (KKS Marburg)

____

Carmen Schade-Brittinger Datum

Leiter der Klinischen Prüfung

____

Prof. Dr. med. Afshin Teymoortash Datum

Biometriker

____

Prof. Dr. rer. nat. Konstantin Strauch Datum

Prüfer

Prüfplanunterschrift

Ich habe dieses Protokoll gelesen und stimme zu, die Studie in Übereinstimmung mit allen Festlegungen dieses Protokolls sowie ICH-GCP, der Deklaration von Helsinki (Version 1996) und des AMG und der GCP-Verordnung durchzuführen.
Außerdem stimme ich zu, sämtliche Informationen bezüglich dieser Studie geheim zu halten

Name des Hauptprüfers (Investigator)
am Prüfzentrum:

____
Name in Druckbuchstaben

____
Unterschrift Datum

Prüfer (Co-Investigator):

____
Name in Druckbuchstaben

____
Unterschrift Datum

Prüfer (Co-Investigator):

____
Name in Druckbuchstaben

____
Unterschrift Datum

1 Liste der Kontaktpersonen

**Leiter der Klinischen Prüfung (LKP)**	Prof. Dr. med. Afshin Teymoortash<br>Leitender Oberarzt der Klinik für Hals-, Nasen- und Ohrenheilkunde der Philipps-Universität<br>Universitätsklinikum Giessen und Marburg,<br>Standort Marburg<br>Deutschhausstr. 3<br>35037 Marburg
**Sponsor**	Philipps-Universität<br>Biegenstrasse 10<br>35037 Marburg
**Bevollmächtigte des Sponsors**	KKS Marburg<br>Karl-von-Frisch-Strasse 4<br>35043 Marburg
**Biometriker:**	Prof. Dr. rer. nat. Konstantin Strauch<br>Institut für Medizinische Biometrie und Epidemiologie der Philipps-Universität Marburg<br>Bunsenstrasse 3<br>35032 Marburg
**Projekt Management:**	Prof. Dr. med. Afshin Teymoortash<br>Leitender Oberarzt der Klinik für Hals-, Nasen- und Ohrenheilkunde der Philipps-Universität<br>Universitätsklinikum Giessen und Marburg,<br>Standort Marburg<br>Deutschhausstr. 3<br>35037 Marburg
**Monitoring**	Koordinierungszentrum für Klinische Studien (KKS)<br>Philipps-Universität Marburg<br>Karl-von-Frisch-Strasse 4<br>35043 Marburg<br>Fax: 06421-28 66 517<br>Tel.: 06421-28 66456
**Serious Adverse Event (SAE) Meldung an:**	Prof. Dr. J.A. Werner<br>Direktor der Klinik für Hals-, Nasen- und Ohrenheilkunde der Philipps-Universität<br>Universitätsklinikum Giessen und Marburg,<br>Standort Marburg<br>Deutschhausstr. 3<br>35037 Marburg<br>Tel.: 06421-5866478<br>Fax.:06421-5866367
**Fax-Randomisierung/Registrierung:**	Koordinierungszentrum für Klinische Studien (KKS)<br>Philipps-Universität Marburg<br>Karl-von-Frisch-Strasse 4<br>35043 Marburg<br>Fax: 06421 - 28 66 517<br>Tel. 06421 - 28 66456

Für weitere Fragen zum Studienablauf kontaktieren Sie bitte:

Medizinische Fragen: Prof. Dr. med. Afshin Teymoortash

Radiologische Untersuchungen:
Dr. med. A. Pfestroff
Klinik für Nuklearmedizin der Philipps-Universität
Universitätsklinikum Giessen und Marburg, Standort Marburg
(Direktor: Prof. Dr. med. T. Behr)
Baldinger Str.
35033 Marburg

Studienmedikation
Botulinum-Toxin A
Allergan Pharmaceuticals Ireland
Castlebar Road
Westport
County Mayo
Ireland

Botulinum-Toxin B
Eisai Manufactoring Knowledge Centre
Mosquito Way, Hatfield
Hertfordshire, AL10 9SN
United Kingdom

2 Inhaltsverzeichnis

1 LISTE DER KONTAKTPERSONEN ... 4
2 INHALTSVERZEICHNIS ... 6
3 PRÜFPLAN-SYNOPSIS ... 8
3.1 STUDIENÜBERSICHT ... 10
4 EINLEITUNG ... 10
5 HINTERGRUND UND RATIONALE ... 12
5.1 VORARBEITEN ... 12
5.2 RATIONALE ... 15
5.3 ZIELSETZUNG ... 16
5.4 POTENTIELLE RISIKEN ... 17
5.5 NUTZEN-RISIKO-ABWÄGUNG ... 17
6 STUDIENZIEL ... 17
PRIMÄRES STUDIENZIEL (ENDPUNKT) ... 17
7 STUDIENDESIGN ... 17
7.1 PATIENTENZAHL ... 17
7.2 BEHANDLUNGSZEITRAUM ... 17
7.3 ZEITPLAN DER STUDIE ... 18
7.4 EINSCHLUSSKRITERIEN ... 18
7.5 AUSSCHLUSSKRITERIEN ... 18
7.6 RANDOMISIERUNG UND REGISTRIERUNG ... 19
7.7 VERBLINDUNG ... 21
7.8 NOTFALLMAßNAHMEN UND ENTBLINDUNG ... 21
8 STUDIENABBRUCH ... 21
8.1 VORZEITIGE BEENDIGUNG DER BEHANDLUNG EINES PATIENTEN ... 21
8.2 VORZEITIGE BEENDIGUNG DER GESAMTSTUDIE ... 22
9 STUDIENMEDIKATION ... 23
9.1 BOTULINUM-TOXIN A UND B ... 23
9.2 BESCHREIBUNG UND WIRKUNGSMECHANISMUS VON BONT ... 23
9.3 HINWEISE FÜR HANDHABUNG VON BOTULINUM-TOXIN ... 24
9.4 RELEVANTE NEBENWIRKUNGEN IM ZUSAMMENHANG MIT BOTULINUM-TOXIN ... 24
9.5 RELEVANTE NEBENWIRKUNGEN IM ZUSAMMENHANG MIT DER BEHANDLUNG ... 25
9.6 HERSTELLER UND KENNZEICHNUNG DER STUDIENMEDIKATION ... 25
9.7 VERGLEICHSMEDIKATION ... 26
9.8 VERPACKUNG UND LABELLING DER STUDIENMEDIKATION UND VERGLEICHSMEDIKATION ... 26
10 STUDIENDURCHFÜHRUNG ... 26
10.1 BASISUNTERSUCHUNGEN VOR THERAPIEBEGINN (SCREENING) ... 26
10.2 UNTERSUCHUNGEN WÄHREND DER BEHANDLUNGSPHASE ... 27
10.3 STUDIENTHERAPIE ... 27
10.4 BEGLEITMEDIKATION UND BEGLEITENDE STRAHLEN- UND CHEMOTHERAPIE ... 28

11 UNTERSUCHUNGSMETHODEN 28

12 SICHERHEIT 28

12.1 DEFINITIONEN ZU UNERWÜNSCHTEN EREIGNISSEN 28
12.2 BEURTEILUNG DER INTENSITÄT 30
12.3 BEURTEILUNG DES KAUSALZUSAMMENHANGS 30
12.4 DOKUMENTATION DER ADVERSE EVENTS (AE) 30
12.5 BEOBACHTUNG / WEITERBEHANDLUNG VON STUDIENTEILNEHMERN MIT ADVERSE EVENTS (AE) 31
12.6 DOKUMENTATION VON SERIOUS ADVERSE EVENTS (SAE) / SERIOUS ADVERSE DRUG REACTIONS (SAR) 31
12.7 PROCEDERE FÜR DIE UNVERZÜGLICHE MELDUNG VON SERIOUS ADVERSE EVENTS (SAE)/ SERIOUS ADVERSE DRUG REACTIONS (SAR) UND UNERWARTETEN SCHWERWIEGENDEN NEBENWIRKUNGEN (SUSAR) 31
12.7.1 Meldung von SAES an den Zweitbewerter/Leiter der klinischen Prüfung 31
12.7.2 Zweitbewertung der Serious Adverse Events(SAE) / Serious Adverse Drug Reactions (SAR) 32
12.8.1 Meldung von SUSARS 33
12.8.2 Form der Meldung von SUSARS 33
12.8.3 Beobachtung von Patienten mit Serious Adverse Event (SAE) und Erstellung eines SAE-Follow-Up 34
12.9 WEITERE GGF. ERFORDERLICHE MELDUNGEN 34
12.10 JAHRESBERICHT (ANNUAL SAFETY REPORT) 35
12.11 BEGLEITERKRANKUNGEN 35
12.12 SCHWANGERSCHAFT 35
12.13 ÜBERDOSIERUNG 36
12.14 ANDERE MEDIZINISCH BESONDERS BEDEUTSAME EREIGNISSE 36

13 ADMINISTRATIVE ASPEKTE 36

13.1 DOKUMENTATION DER DATEN IM CRF 36
13.2 ORIGINALDATEN UND ORIGINALDOKUMENTE 37
13.3 SICHERSTELLUNG DER DATENQUALITÄT, MONITORING, AUDIT 37
13.4 PRÜFARZTORDNER (INVESTIGATOR SITE FILE) / ARCHIVIERUNG DES PRÜFARZTORDNERS 38
13.5 DATENMANAGEMENT UND ARCHIVIERUNG 38
13.6 DATENSCHUTZ 39

14 ETHISCHE UND RECHTLICHE ASPEKTE 39

14.1 VERANTWORTLICHKEIT DES HAUPTPRÜFERS (INVESTIGATOR) AM PRÜFZENTRUM 39
14.2 PATIENTENINFORMATION UND PATIENTENEINWILLIGUNG 39
14.3 PATIENTENVERSICHERUNG 40
14.4 BEWERTUNG DURCH DIE ETHIKKOMMISSION NACH AMG 40
14.5 BEHÖRDLICHE GENEHMIGUNGS- UND ANZEIGEVERFAHREN NACH AMG 41
14.6 PROTOKOLL-AMENDMENT 41
14.7 NOTWENDIGE STUDIENDOKUMENTE VOR STUDIENBEGINN 41

15 STATISTISCHE METHODEN 42

16 VERANTWORTLICHKEIT 43

17 LITERATUR 44

18 ANHÄNGE 46

18.1 ANHANG 1: BEHANDLUNGSPROTOKOLLE 46
RADIOCHEMOTHERAPIEPROTOKOLL: 47
Chemotherapie 47

3 Prüfplan-Synopsis

**Titel der Studie**	Funktionsszintigraphische Untersuchungen zur Überprüfung der radioprotektiven Wirksamkeit intraglandulär applizierten Botulinum-Toxins (BoNT) auf die Gl. submandibularis bei Patienten mit einem Plattenepithelkarzinom der oberen Luft- und Speisewege
**Sponsor**	s.o.
**Leiter der Klinischen Prüfung (LKP)**	Prof. Dr. med. Afshin Teymoortash<br>Leitender Oberarzt der Klinik für Hals-, Nasen- und Ohrenheilkunde der Philipps-Universität Universitätsklinikum Giessen und Marburg,<br>Standort Marburg<br>Deutschhausstr. 3<br>35037 Marburg
**Studienzentren/-länder**	1 Zentrum, Deutschland
**Studiendesign / Studienphase und Indikation**	• Vierarmige monozentrische, randomisierte, doppelblinde Phase I-Studie<br>• Strahlensialadenitis
**Prüfmedikation (IMP)**	• BoNT/A und BoNT/B
**Menge und Art der Dosierung**	• 6 Pat BoNT/A in einer Dosierung von 15 U<br>• 6 Pat BoNT/A (15U) und BoNT/B (750U)
**Behandlungsart und- dauer pro Patient**	• Insgesamt einmalige Injektion vor Beginn der geplanten Strahlentherapie<br>• Dauer der Strahlentherapie ca. 7 Wochen
**Studiendesign**	Bei Patienten mit einem Plattenepithelkarzinom der oberen Luft- und Speisewege soll eine primäre konventionelle Radiochemotherapie der Halsweichteile durchgeführt werden, wobei die Gl. submandibularis beidseits im Strahlenfeld liegen. BoNT soll vor der geplanten Strahlentherapie bei allen Patienten je nach Ergebnis der Randomisierung in die rechte oder linke Gl. submandibularis appliziert werden, Applikation von gleichem Volumen NaCl erfolgt in die gegenseitige Gl. submandubularis, als Kontrolldrüse. Funktionsprüfung der Gl. submandibularis beidseits erfolgt durch Speichel-drüsenszintigraphie vor bzw. 4 Monate nach Beendigung der Strahlentherapie.
**Geplante Anzahl Patienten**	12 Patienten

**Einschlusskriterien**	• Patienten mit einem Plattenepithelkarzinom der oberen Luft- und Speisewege<br>• Schriftliche Einwilligung zur Teilnahme an der Studie<br>• Kognitive und physische Fähigkeit des Probanden, den Versuchsablauf zu verstehen und einzuhalten.<br>• Alter ≥ 40 Jahre<br>• Geschlecht: weiblich und männliche Teilnehmer<br>• Frauen im gebärfähigen Alter ab 18 Jahren müssen eine der folgenden Methoden zur Empfängnisverhütung anwenden: orales Kontrazeptivum und Intrauterinpessar (Spirale), seit mindestens einem Monat vor Studienbeginn, Barriere mit Spermizid (Kondom oder Diaphragma), Tubenligatur. Als Frauen im gebärfähigen Alter werden alle Frauen angesehen, deren letzte Menstruation nicht länger als 1 Jahr zurückliegt<br>• Keine Funktionseinschränkungen und Seiten-Unterscheide im Rahmen der initialen Speicheldrüsenfunktionsszintigraphie
**Ausschlusskriterien**	• Z.n. Exstirpation der Gl. submandibularis<br>• anamnestisch bekannte Erkrankungen der Speicheldrüsen<br>• regelmäßige Einnahme von Medikamenten, die die Funktion der Speicheldrüsen beeinflussen<br>• Z.n. vorheriger Bestrahlung der Halsweichteile<br>• Schwangerschaft<br>• Stillzeit<br>• Irenat-Therapie<br>• Überempfindlichkeit gegenüber Botulinum-Toxin oder einem der sonstigen Bestandteile
**Zielkriterium**	Hauptzielkriterium<br>Vorher-Nachher-Unterschied der Differenz zwischen der prozentualen Aufnahme (Uptake) des Radionuklids durch die mit BoNT behandelte und die gegenseitige Gl. submandibularis<br>Nebenzielkriterium<br>Vorher-Nachher-Unterschied der Differenz zwischen der Ejektionsfraktion nach Gabe eines Reizgetränks für die mit BoNT behandelte und die gegenseitige Gl. submandibularis
**Statistische Methoden**	Wilcoxon signed rank Test, Mann-Whitney-U-Test, deskriptive Auswertung
**Studiendauer**	ca. 7-8 Monate pro Patient<br>ca. 23 Monate gesamt

3.1 Studienübersicht

Studienspezifische Untersuchungen und Procedere	Screening	Pre-treatment	Visiten Woche 1-7	Post-treatment
Ein-/Ausschlusskriterien	x			
Aufklärung	x			
Einwilligungserklärung	x			
Schwangerschaftstest	x			
Randomisierung/Registrierung	x			
Speicheldrüsenfunktions-prüfung/szintigraphie *	x			x[1]
BoNT Injektion *		X[2]		
Strahlentherapie *			Woche 1,2,3,4,5,6,7	
Chemotherapie *			Woche 1 ,4, 7	
Supportivtherapie *			x	

*analog Anhang 1 zum Studienprotokoll

1) 4 Monate nach Strahlentherapie

2) 2 Wochen vor Bestrahlung

4 Einleitung

Karzinome der oberen Luft- und Speisewege machen einen Anteil von ca. 5% aller Erstmanifestationen maligner Tumore aus. Ihre Inzidenz wird in der Bundesrepublik Deutschland auf ca. 15000 Neuerkrankungen im Jahr geschätzt. Die malignen Tumore der oberen Luft- und Speisewege werden in der Regel chirurgisch, radio(chemo)therapeutisch oder durch eine Kombination der genannten Verfahren behandelt. So kommt der Strahlentherapie eine zentrale Bedeutung im Behandlungskonzept von Malignomen der genannten Region zu.

Dem positiven Effekt der Bestrahlung auf den Tumor steht die Schädigung des umgebenden Normalgewebes gegenüber. In der akuten Phase der Radiotherapie entwickelt sich v.a. eine in der Intensität unterschiedlich ausgeprägte Dermatitis und Mukositis. Im weiteren Verlauf der Bestrahlung und in der postradiogenen Phase entwickelt sich häufig eine von den Patienten unangenehm empfundene Mundtrockenheit. Diese Xerostomie bleibt als Spätfolge der radiogenen Schädigung der kleinen und großen Speicheldrüsen auch nach Abschluss der Strahlentherapie in mehr oder minder starkem Maße lebenslang bestehen. Die durch

Strahleneinwirkung bedingte Speicheldrüsenentzündung und damit verbundene Speicheldrüsenfunktionsstörung führt unter anderem zur Dysphagie und zum Gewichtsverlust bei den ohnehin in ihrer Schluckfunktion beeinträchtigten Tumorpatienten. Des Weiteren sind diese Patienten durch fehlende Speichelwirkung anfällig für Infektionskrankheiten der Schleimhäute der oberen Luft- und Speisewege, der Zähne und des Zahnhalteapparates, des Kieferknochens bis hin zu einer Osteoradionekrose. Die strahleninduzierte Sialadenitis führt zu einer erheblichen Reduktion der Lebensqualität der betroffenen Patienten [1,2].

Aufgrund der großen klinischen Relevanz dieser Erkrankung besteht ein dringender Bedarf an radioprotektiven Substanzen für Kopfspeicheldrüsen. Trotz vieler Versuche und Bemühungen an menschlichen Speicheldrüsen und im Tiermodell sind bisher allerdings keine wesentlichen Fortschritte zur medikamentösen Vorbeugung der strahleninduzierte Sialadenitis erzielt worden. Durch die momentan verfügbaren konservativen Therapiemaßnahmen wie einer erhöhten Flüssigkeitszufuhr, einer intensiven Mundhygiene, antimykotischer und antibakterizider sowie ggfs. immunsuppressiver und kortikoidhaltiger Therapieregime können die mit einer Xerostomie einhergehenden Beschwerden nicht geheilt und in den meisten Fällen nur unwesentlich gelindert werden. In dem Bewusstsein um die Auswirkungen einer strahleninduzierten Xerostomie wurden vor diesem Hintergrund sogar Bemühungen unternommen, eine oder beide Glandulae submandibulares temporär zu explantieren. Neben dem chirurgischen Aufwand und der kosmetisch relevanten Narbenbildung wird hierbei auch das Risiko einer akzidentellen Verletzung des Ramus marginalis des N. facialis sowie eine Schädigung des N. lingualis bewusst in Kauf genommen [3].

Eine mögliche Rolle der sog. intensitätsmodulierten Strahlentherapie (IMRT) wird aktuell intensiv zur Prävention der Strahlensialadenitis diskutiert. Dieses Verfahren ermöglicht bei unregelmäßigen Zielvolumina, bei denen Risikostrukturen in Konkavitäten des Zielvolumens eingebettet sind, eine streng an das Zielvolumen angepasste Dosisverteilung zu generieren, die einerseits hohe Dosen im Zielvolumen applizieren lässt und andererseits die Toleranzdosen der Risikostrukturen respektieren hilft. So konnte nach IMRT der Kopf-Hals-Karzinome zwar eine höhere Speichelflußrate der Gl. parotis im Vergleich zu den konventionell bestrahlten Drüsen erreicht werden, allerdings ohne signifikante Besserung der Xerostomie-Symptomatik der bestrahlten Patienten [4]. Zur Strahlen-Prävention der Speicheldrüsen ist der Schutz der muzin-sezernierenden Gl. submandibularis mit der höchsten Rate an Ruhesekretion von Bedeutung. Mit IMRT ist eine suffiziente

Dosisreduktion im submandibulären Bereich aufgrund der engen Beziehung der Gl. submandibularis zu den erstdrainierenden Lymphknoten, welche bei der überwiegenden Mehrheit der Kopf-Hals-Karzinome submandibulär lokalisiert sind, nicht realisierbar [5].

5 Hintergrund und Rationale

Im Rahmen einer Voruntersuchung (siehe unten, Vorarbeit 1) wurde festgestellt, dass es während der medikamentösen Denervierung der Speicheldrüsen durch BoNT gleichzeitig zu einer temporären Azinusatrophie kommt [6]. Vor diesem Hintergrund ist von Interesse, dass der jeweilige Funktionszustand des Azinusgewebes und hier insbesondere die Menge der Sekretgranula in den Drüsenazini ein wichtiger pathogenetischer Faktor für die strahleninduzierte Sialadenitis darstellt. Basierend auf diesen Kenntnissen wurde untersucht, ob es bei einer vor der Bestrahlung durchgeführten intraglandulären Injektion von BoNT zu einer passageren Drüsenatrophie für die Zeitdauer der Strahlentherapie kommt, welche die Strahlensensibilität der Drüsenazini deutlich reduzieren könnte.

Die genannte Hypothese bildete die Grundlage für eine weitere tierexperimentelle Arbeit zur Abklärung einer möglichen radioprotektiven Wirksamkeit des BoNT auf Speicheldrüsen der Wistar-Ratte [7]. In dieser Untersuchung (siehe unten, Vorarbeit 2) konnte morphologisch und funktionsszinigraphisch festgestellt werden, dass die vor der Strahlentherapie ausgeführte intraglanduläre Applikation von BoNT die strahleninduzierte Drüsenschädigung signifikant reduziert.

Mit der vorliegend beantragten Untersuchung soll die radioprotektive Wirksamkeit des BoNT im Rahmen einer Phase-I-Studie bei Patienten mit einem Plattenepithelkarzinom der oberen Luft- und Speisewege, die zur Behandlung ihres Karzinoms eine primäre Strahlentherapie erhalten sollen, analysiert werden.

5.1 Vorarbeiten

1) Untersuchungen zur morphologischen Veränderungen der Speicheldrüsen nach Applikation von Botulinum-Toxinen [6]

Fragestellung: Pharmakologische Denervierung der Speicheldrüsen durch intraglanduläre Applikation von BoNT hemmt die Acetylcholinausschüttung an der neuroglandulären Synapse und führt zu einer Reduktion des Speichelflusses. Gleichzeitig kommt es zu einem Anstieg der Konzentration bestimmter Speichelkomponenten [8]. Nach Berechnung des Output/min dieser Speichelkomponenten bezogen auf die Speichelflussrate/min wurde

allerdings deutlich, dass kein Unterschied der Werte vor und nach Toxinapplikation bestand. Daraus konnte gefolgert werden, dass die Proteinsekretion der Kopfspeicheldrüsen nach BoNT-Applikation nicht wesentlich beeinflusst wird. In dieser und in anderen Studien an Mensch und Ratte wurden die genannten Effekte mit der dualen Innervation der Speicheldrüsen erklärt: Die überwiegend intakte adrenerge Komponente und isolierte cholinerge Inhibition durch BoNT bewirken eine Reduktion der Speichelmenge, lassen die Proteinproduktion aber weitgehend unbeeinflusst [9].

Die hierzu existierenden Daten lassen allerdings keine Schlüsse über die genaue Wirkung der BoNT auf die Speichelproteinproduktion und Funktion der Speicheldrüsen nach intraglandulärer Injekion zu. Weiterhin ist unklar, ob die chemische Denervierung gleichzeitig zu einer temporären Azinusatrophie der Drüsen führt. Ellies et al. [10] führten hierzu morphometrische Studien an der Glandula submandibularis der Ratte nach BoNT A Behandlung durch. Ihre Messungen ergaben keinen signifikanten Unterschied zwischen toxin- und kochsalzinjizierten Drüsen in Zellzahl und -größe. Die morphometrischen Auswertungen in dieser Studie wurden allerdings limitiert auf die Zählung der Nuclei der serösen Acinuszellen. Zusätzlich wurde von den Autoren der genannten Studie keine standarisierte Methode zur Fixation des Gewebes vor den morphometrischen Messungen verwendet, um die Unterschiede in der Schrumpfung des Gewebes während der Fixation zu minimieren.

Exakte Untersuchungen zum Verständnis der morphologischen und ultrastrukturellen Veränderungen nach BoNT Behandlung der Speicheldrüsen fehlen. Ziel dieser Voruntersuchung war es, die morphologischen und funktionellen Veränderungen in den Speicheldrüsen nach Applikation von BoNT A und B zu analysieren und festzustellen, ob eine temporäre Azinusatrophie der chemodenervierten Drüse stattfindet.

Studiendesign: Es wurden die Gewebeproben der Unterkieferspeicheldrüsen (Gl. submandibularis) von 18 Wistar-Ratten nach intraglandulärer Injektion von Botulinum-Toxin A (5U), B (250U) und Kombination von A und B histologisch, immunhistochemisch sowie elektronenmikroskopisch analysiert. Die kontralaterale Kontroll-Drüse wurde jeweils mit Kochsalzlösung behandelt.

Ergebnisse: Nach Untersuchung der Drüsen nach 14 Tagen zeigte sich eine signifikante Reduktion des Drüsengewichtes nach Injektion von BoNT (P=0,016). In morphologisch-morphometrischen und ultrastrukturellen Analysen der Drüsenzellen insbesondere der

Sekretgranula mittels Semidünnschnitten und elektronenmikroskopischen Analysen des Drüsengewebes konnten eindeutige Zeichen der Atrophie der Azinuszellen gefunden werden. Diese waren am deutlichsten in den mit BoNTA und B in Kombination behandelten Drüsen zu sehen. Durch morphometrische Untersuchungen der Drüsenazini konnte eine signifikante Reduktion der Azinuszellfläche nach Injektion von Botulinum-Toxin festgestellt werden (P=0,031). Weiterhin zeigten Western-Blot-Analysen und immunhistochemische Amylasefärbungen des Drüsengewebes eine reduzierte Amylaseproduktion in den mit BoNT behandelten submandibulären Drüsen, im Vergleich zu den Kontrolldrüsen.

Diskussion und Schlussfolgerung: SNAP-23(25) (BoNT A) und VAMP (BoNT B) sind Enzyme, die an der Freisetzung von Acetylcholin an der präsynaptischen Membran von parasympathischen Nervenendigungen beteiligt sind. Somit führen BoNT (A, B) durch die Spaltung dieser Enzyme zu einer temporären Chemodenervation am Zielorgan. Die Exocytose in exokrinen Zellen ist allerdings ebenfalls SNARE-Protein abhängig [11,12]. In Speicheldrüsenazinuszellen konnte VAMP-2 an der Membran sekretorischer Granula nachgewiesen werden [13]. Im Bereich der Azini der Gl. parotis wurden Syntaxin-4 und SNAP-23 sowie weitere Proteine an der Plasmamembran gefunden [14]. Die spezifische Rolle dieser Proteine bei der Sekretion der Granula ist noch nicht in allen Details bekannt. Sie könnten allerdings spezifische v-SNAREs an der apikalen Plasmamembran von Azinuszellen repräsentieren [15]. Die Inhibition der cholinergen parasympathischen Innervation an der neuroglandulären Synapse und speziell die Spaltung der an der Exocytose beteiligten SNAREs in Acinuszellen durch BoNT scheinen die Morphologie und die Funktion der Drüsen zu beeinflussen.

Ultrastrukturelle Analysen der Zellorganellen und Sekretgranula verdeutlichten eindeutig eine Atrophie der Azini unterschiedlicher Ausprägung bei verschiedenen Toxin-Subtypen. Bei der kombinierten Applikation von BoNT A und B konnte eine höhergradige Atrophie des Drüsenparynchyms im Vergleich zu der isolierten Gabe von Botulinum-Toxin A oder B festgestellt werden.

Die dargestellten Ergebnisse weisen darauf hin, dass entgegen der bisherigen Annahme durch BoNT nicht nur aufgrund der Inhibition der cholinergen Innervation die Speichelsekretion beeinflusst wird, sondern offenbar die Inhibition der SNARE-Proteine der Azini zu deren Funktionsstörung führt.

2) Untersuchungen zur radioprotektiven Wirksamkeit des intraglandulär applizierten Botulinum-Toxins auf die Gl. submandibularis der Wistar-Ratte [7].

Fragestellung: Der Strahlentherapie kommt eine zentrale Bedeutung im Behandlungskonzept von Karzinomen der oberen Luft- und Speisewege zu. Xerostomie bleibt als Spätfolge der radiogenen Schädigung der Speicheldrüsen nach Abschluss der Strahlentherapie lebenslang bestehen und führt zu einer erheblichen Reduktion der Lebensqualität der betroffenen Patienten. Durch die z.Z. verfügbaren Therapiemaßnahmen können die mit einer Xerostomie einhergehenden Beschwerden nur unwesentlich gelindert werden. In der vorliegenden Untersuchung sollte erstmals die radioprotektive Wirkung von Botulinum-Toxin (BoNT) in einem Tiermodell geprüft werden.

Studiendesign: Nach intraglandulärer Injektion von BoNT in die Gl. submandibularis der Wistar-Ratte (n=8) und Injektion eines gleichen Volumens Kochsalzlösung in die kontralaterale Kontroll-Drüse erfolgte die Bestrahlung der behandelten Drüsen mit einer Einzeldosis von 20 Gy. Funktionsszintigraphische und morphologische Untersuchungen schlossen sich drei Monate nach Bestrahlung an.

Ergebnisse: Der szintigraphische Vergleich der Drüsen zeigte eine signifikant höhere gland-to-background ratio in den mit BoNT-behandelten Drüsen (P=0.008). In Vitro Messungen bestätigten die signifikant höhere Menge an Radioaktivität in der BoNT-Gruppe (P=0.015). Histologische Untersuchungen zeigten einen deutlich besseren Erhalt des Drüsenparenchyms nach BoNT-Applikation. Die mit BoNT-behandelten Drüsen wurden histologisch im Stadium 1 der strahleninduzierten Sialadenitis eingestuft, während sich die Kontroll-Drüsen im Stadium 2 und 3 befanden.

Schlussfolgerung: In dieser Pilotstudie konnte gezeigt werden, dass die intraglanduläre Applikation von BoNT vor der Bestrahlung signifikant die strahleninduzierte Drüsenschädigung reduzieren kann.

5.2 Rationale

Aus den experimentellen Daten ergibt sich, dass der ausschlaggebende pathogenetische Faktor für eine strahleninduzierte Sialadenitis die direkte radiogene Einwirkung auf das Drüsengewebe ist. Strahlendosis und Zeitdauer der Strahleneinwirkung stehen in direkter

Relation zum Schweregrad der Schädigung des Drüsengewebes. Ein weiterer wichtiger pathogenetischer Faktor stellt der jeweilige Funktionszustand des Azinusgewebe dar, insbesondere die Menge der Sekretgranula in den Drüsenazini. Eine mögliche Erklärung für die Bedeutung des Granulagehaltes der Azini für die Auslösung des Strahlenschadens ergibt sich daraus, dass die membranbegrenzten Sekretgranula reichlich Schwermetalle enthalten. Diese besitzen die Fähigkeit zur Bildung von Redoxsystemen mit einer erhöhten Sensitivität gegenüber ionisierenden Strahlen [16,17].

Mit BoNT steht eine klinisch erprobte und gut verträgliche Substanz zur Verfügung, welche seit Jahren zunehmend zur Behandlung verschiedener Erkrankungen der Kopfspeicheldrüsen wie Sialorrhoe, Speichelfistel und Frey-Syndrom erfolgreich routinemäßig eingesetzt wird. Durch intraglanduläre Injektion von BoNT kommt es zu einer passageren Denervierung der Drüse mit folgender Reduktion der Speichelsekretion. Im Rahmen der oben erwähnten Voruntersuchung (Vorarbeit 1) wurde festgestellt, dass es während der medikamentösen Drüsendenervierung gleichzeitig zu einer temporären Azinusatrophie kommt.

In der bereits durchgeführten Untersuchung (Vorarbeit 2) konnte bestätigt werden, dass es bei einer vor der Bestrahlung durchgeführten intraglandulären Injektion von BoNT zur einer passageren Drüseninvolution für die Zeitdauer der Bestrahlung kommt, welche die Strahlensensibilität der Drüsenazini deutlich zu reduzieren vermag. Es konnte ein signifikant besserer Erhalt des Drüsenparenchyms nach Bestrahlung bei mit BoNT behandelten Drüsen sowohl morphologisch als auch szintigraphisch festgestellt werden.

5.3 Zielsetzung

Die geplante Phase I-Studie verfolgt folgende Zielsetzungen:

Auf dieser Grundlage und zur weiteren Abklärung der genannten Hypothese ist das Ziel des klinischen Behandlungsversuches die Funktionsprüfung der Speicheldrüsen durch quantitative Sialoszintigraphie zur Erlangung von ersten Erkenntnissen zur möglichen radioprotektiven Wirkung, Sicherheit und Dosierung des Botulinum-Toxins auf die Gl. submandibularis bei Patienten mit einem Plattenepithelkarzinom der oberen Luft- und Speisewege. Bevor dieses in Detail hinterfragt wird, gilt es in einer Phase-I-Studie die ersten Erkenntnisse zu gewinnen.

5.4 Potentielle Risiken

Siehe 9.4 und 9.5!

5.5 Nutzen-Risiko-Abwägung

Durch die momentan verfügbaren konservativen Therapiemaßnahmen wie einer erhöhten Flüssigkeitszufuhr, einer intensiven Mundhygiene, antimykotischer und antibakterizider Therapie können die mit einer Xerostomie einhergehenden Beschwerden nicht geheilt und in den meisten Fällen nur unwesentlich gelindert werden. Diese Beschwerden führen zu einer lebenslang anhaltenden signifikanten Reduktion der Lebensqualität der Tumorpatienten. Aufgrund der vielversprechenden Ergebnisse der Voruntersuchungen und sehr geringe Belastung der Medikamenten-Applikation, wäre eine Therapie mit diesem Medikament bezüglich Risiko-Nutzen-Abwägung gerechtfertigt.

6 Studienziel

Primäres Studienziel (Endpunkt)

Sicherheit, Dosierung und mögliche radioprotektive Wirkung von BoNT auf Speicheldrüsen beim Patienten.

7 Studiendesign

Vierarmige monozentrische, randomisierte, doppelblinde Phase I-Studie, die nur in 1 Prüfzentrum (Deutschland) durchgeführt werden soll.

7.1 Patientenzahl

Für die geplante Studie sind 12 Patienten mit einem Plattenepithelkarzinom der oberen Luft- und Speisewege vorgesehen. Die Untersuchung ist nicht geschlechtsspezifisch und es sollen Frauen wie Männer untersucht werden. Da die überwiegende Mehrheit der Patienten (weit über 95%) mit dieser Erkrankung über 40 Jahre alt sind, wird in der geplanten Untersuchung diese Altergruppe berücksichtigt.

7.2 Behandlungszeitraum

Die Strahlentherapie beträgt etwa 7 Wochen, die Funktionsprüfung der Gl. submandibularis beidseits erfolgt anschließend durch Speicheldrüsenszintigraphie etwa 4 Monate nach Beendigung der Strahlentherapie.

7.3 Zeitplan der Studie

Voraussichtlicher Beginn der Studie: Sobald das positive Votum zur Durchführung der Studie vorliegt.

Voraussichtliches Ende der Studie: Nachdem der letzte Patient die Kontroll-Szintigraphie absolviert hat.

Dauer der Studie pro Patient : ca. 7-8 Monate
Dauer der Gesamtstudie: ca. 23 Monate

7.4 Einschlusskriterien

Bei Patienten mit einem Plattenepithelkarzinom der oberen Luft- und Speisewege wird aufgrund der Tumorausdehnung und des Ausmaßes der lymphogenen Metastasierung eine primäre konventionelle Radiochemotherapie der Halsweichteile durchgeführt, wobei die Gl. submandibularis beidseits im Strahlenfeld liegen und mit der gleichen Strahlendosis bestrahlt werden. Dieses Vorgehen entspricht der klinischen Routine.

- Patienten mit einem Plattenepithelkarzinom der oberen Luft- und Speisewege
- Schriftliche Einwilligung zur Teilnahme an der Studie
- Kognitive und physische Fähigkeit des Probanden, den Versuchsablauf zu verstehen und einzuhalten.
- Alter ≥ 40 Jahre
- Geschlecht : weibliche und männliche Teilnehmer
- Frauen im gebärfähigen Alter ab 18 Jahren müssen eine der folgenden Methoden zur Empfängnisverhütung anwenden: orales Kontrazeptivum und Intrauterinpessar (Spirale), seit mindestens einem Monat vor Studienbeginn, Barriere mit Spermizid (Kondom oder Diaphragma), Tubenligatur. Als Frauen im gebärfähigen Alter werden alle Frauen angesehen, deren letzte Menstruation nicht länger als 1 Jahr zurückliegt
- Keine Funktionseinschränkungen und Seiten-Unterscheide im Rahmen der initialen Speicheldrüsenfunktionsszintigraphie

7.5 Ausschlusskriterien

- Z.n. Exstirpation der Gl. Submandibularis
- anamnestisch bekannte Erkrankungen der Speicheldrüsen

- regelmäßige Einnahme von Medikamenten, die Funktion der Speicheldrüsen beeinflussen
- Z.n. vorheriger Bestrahlung der Halsweichteile
- Schwangerschaft
- Stillzeit
- Irenat-Therapie.
- Überempfindlichkeit gegenüber Botulinum-Toxin oder einem der sonstigen Bestandteile

7.6 Randomisierung und Registrierung

Nach ausreichender Aufklärung und Vorlage einer schriftlichen Einverständniserklärung müssen alle geeigneten Patienten per Fax randomisiert und registriert werden. Es ist die Aufgabe des Prüfarztes sicherzustellen, dass alle Ein- und Ausschlusskriterien überprüft, erfüllt und dokumentiert sind. Das ausgefüllte und unterschriebene Formblatt (im CRF-Ordner) wird per Fax an folgende Adresse gesendet:

Koordinierungszentrum für Klinische Studien (KKS)
Philipps-Universität Marburg
Karl-von-Frisch-Strasse 4
35043 Marburg
Fax (06421) 28 66517
Tel. (06421) 28 66541

Die Randomisierung und Registrierung eines Patienten ist zu ausschließlich folgenden Zeiten möglich:

Montags – Donnerstag: 8:00 Uhr – 16:00
Freitag: 8:00 Uhr – 14:00 Uhr

<u>Behandlungsgruppen:</u>

1: BoNT/A (15U) Gl.submand. rechts, NaCl Gl. submand. links
2: BoNT/A (15U) Gl.submand. links, NaCl Gl. submand. rechts
3: BoNT/A (15U) und BoNT/B (750U), Gl. submand. rechts, NaCl Gl. submand. links
4: BoNT/A (15U) und BoNT/B (750U), Gl. submand. links, NaCl Gl. submand. rechts

Sechs zufällig ausgewählte Patienten sollen mit BoNT/A in einer Dosierung von 15 U behandelt werden, die anderen sechs Patienten mit BoNT/A (15U) und BoNT/B (750U). In jeder der beiden Dosisgruppen wird das Medikament bei je drei Patienten in die rechte bzw. linke Gl. submandibularis injiziert, wobei die Seite wiederum zufällig ausgewählt wird. Applikation von gleichem Volumen NaCl erfolgt bei allen Patienten in die gegenseitige Gl. submandubularis, als Kontrolldrüse für eine spätere intraindividuelle Vergleichbarkeit. Das Gesamt-Volumen des applizierten Medikaments bzw. NaCl im Bereich der Gl. submandibularis beträgt jeweils 0.8 ml. Um die Patienten den verschiedenen Gruppen zuzuweisen, wird für jeden Patienten eine Zufallszahl mit Wert 1, 2, 3 oder 4 gezogen. Dabei bedeutet ’1’ die Zuteilung des Patienten zur BoNT/A-Gruppe mit Injektion des Medikaments in die rechte Gl. submandibularis, ’2’ BoNT/A-Injektion in die linke Gl. submandibularis, ’3’ BoNT/A- und B-Injektion in die rechte und ’4’ BoNT/A- und B-Injektion in die linke Gl. submandibularis. Die Patienten werden balanciert randomisiert, so daß jedem der vier Studienarme drei Patienten zugewiesen werden. Auf diese Weise wird ein möglicher Seiteneffekt (rechts vs. links) auf das Behandlungsergebnis so weit wie möglich ausgeglichen. Der verantwortliche Biometriker (KS) erstellt die Randomisierungsliste und übermittelt sie vor Beginn der Studie an das KKS, welches die Randomisierung im Rahmen der Patientenregistrierung durchführt.

Nach erfolgter Zuteilung der Patienten entsprechend der Randomisierungsliste zu einer der Gruppen 1-4 erhält jeder Studienteilnehmer noch eine zufällig bestimmte, eindeutige vierstellige KKS-interne Nummer, anhand welcher der zugehörige Notfallumschlag identifiziert werden kann (siehe unten, Notfallmaßnahmen und Entblindung). Die Zuordnung dieser Nummer zum Studienarm des Patienten ist den Prüfärzten ohne Öffnung des Umschlags nicht bekannt, so dass die Verblindung und das Concealment of allocation gewährleistet sind. Das KKS teilt dem verantwortlichen Biometriker (KS) die Zuordnung der KKS-internen Nummer zur Patientenidentifikationsnummer und damit zum Studienarm im Rahmen der statistischen Auswertung erst zum spätestmöglichen Zeitpunkt mit und übermittelt die Daten lediglich unter Angabe der KKS-internen Nummer. Auf diese Weise können Entscheidungen darüber, wie möglicherweise auftretende Protokollverletzungen oder fehlende Daten bei der Auswertung gehandhabt werden sollen, unabhängig von der Kenntnis über die Zugehörigkeit zum Studienarm getroffen werden.

7.7 Verblindung

Sowohl die behandelnden und untersuchenden Ärzte (Injektion von BoNT bzw. Kochsalzlösung sowie szintigraphische Untersuchung) als auch die Patienten sind verblindet; es handelt sich also um eine Doppelblindstudie.
Der Prüfarzt erhält nach der Randomisierung lediglich die eindeutige KKS-interne Nummer per Fax mitgeteilt.

7.8 Notfallmaßnahmen und Entblindung

Die Verblindung darf nur aufgehoben werden, wenn im Notfall eine Identifizierung der Prüfmedikation tatsächlich erforderlich ist.
Das Zentrum erhält zu Studienbeginn verschlossene Briefumschläge (gesammelt für alle Studienteilnehmer), auf denen außen die eindeutige KKS-interne Nummer vermerkt ist, und welche die genauen Angaben für die Therapieform enthalten. Nach erfolgter Randomisation muss der Prüfarzt sofort nach Benachrichtigung die Patientenidentifikationsnummer auf dem Briefumschlag notieren. Der Monitor überprüft bei seinen Besuchen alle Briefumschläge auf Unversehrtheit. Es ist zu dokumentieren, von wem, wann und warum das Notfallkuvert geöffnet wurde. Um den doppelblinden Charakter der Studie aufrecht zu erhalten, darf die Information über die Studienmedikation des betreffenden Patienten nur dem zuständigen Apotheker mitgeteilt werden, damit er dem Prüfarzt die entsprechende Prüfmedikation überreichen kann.

8 Studienabbruch

8.1 Vorzeitige Beendigung der Behandlung eines Patienten

Die protokollgemäße Behandlung eines Studienpatienten kann abgebrochen werden wenn:

- ein Adverse Event (AE), Serious Adverse Event (SAE) oder lebensbedrohliches Ereignis auftritt, das eine protokollgemäße Fortsetzung der Therapie nicht mehr zulässt.
- der Prüfarzt im Interesse des Patienten aus medizinischen Gründen entscheidet, die protokollgemäße Behandlung des Patienten abzubrechen.
- mangelnde Compliance des Patienten vorliegt.

Die protokollgemäße Behandlung eines Studienpatienten muss abgebrochen werden wenn:

- der Patient das Einverständnis zur Teilnahme an der Studie zurückzieht. Der Patient hat das Recht, jederzeit aus der Studie auszuscheiden, ohne dass nachteilige Folgen für die weitere Behandlung entstehen.
- eine Schwangerschaft auftritt. Der Verlauf sollte bis zum Ende der Schwangerschaft dokumentiert werden.

Wurde die protokollgemäße Behandlung eines Patienten durch den Prüfarzt abgebrochen, so hat dieser den Patienten über den vorzeitigen Abbruch zu informieren. Die im Studienprotokoll festgelegten Untersuchungen bei Therapieabschluss sind durchzuführen und der Studienabbruch ist in der Krankenakte des Patienten sowie im CRF zu dokumentieren.

Für den Fall, dass der Patient den Studienabbruch veranlasst hat, können die vor dem Studientherapieabbruch erhobenen Daten ausgewertet werden. Für die protokollgemäße Nachbeobachtung (Follow Up) ist das schriftliche Einverständnis einzuholen. Stimmt der Patient der im Studienprotokoll festgelegten Nachbeobachtungsphase zu, können die hierbei erhobenen Daten ebenfalls dokumentiert und ausgewertet werden.

Generell soll jeder Versuch unternommen werden, Patienten, die die Behandlung vorzeitig beenden, bis zum Abschluss der Studie weiter zu beobachten und zu dokumentieren.

8.2 *Vorzeitige Beendigung der Gesamtstudie*

Ein vorzeitiger Abbruch der Gesamtstudie ist in folgenden Fällen vorgesehen:

- Auftreten neuer wissenschaftlicher Erkenntnisse während der Laufzeit der Studie, die eine Fortsetzung der Studie aus ethischen Gründen nicht erlauben würden.
- Auftreten von einer unerwarteten Anhäufung von Adverse Events (AE) / Serious Adverse Events (SAE).
- Nichterreichen der geplanten Patientenzahl innerhalb des Studienzeitraums.

Die Entscheidung, ob ein relevanter Grund für einen vorzeitigen Abbruch der Studie vorliegt, wird von der Studienleitung und dem Sponsor getroffen.

9 Studienmedikation

9.1 Botulinum-Toxin A und B

9.2 Beschreibung und Wirkungsmechanismus von BoNT

Das Botulinum-Toxin (BoNT) ist ein Proteinkomplex, der sich aus dem Neurotoxin (150 kDa), den Hämagglutinen und einem nicht toxischen Protein zusammensetzt. BoNT wird von dem grampositiven Anaerobier Clostridium botulinum produziert. Es werden sieben Serotypen des Botulinum-Toxins unterschieden, deren unterschiedliche Neurotoxine im terminalen Axon cholinerger Nerven den Fusionskomplex, aus den drei Proteinen VAMP (vesikelassoziiertes Membranprotein), SNAP-25 (synaptosomenassoziiertes Protein) und Zielmembranprotein Syntaxin, inaktivieren. Durch diese Blockade wird die Ausschüttung der Acetylcholin-gefüllten Vesikel in den synaptischen Spalt verhindert [18].

Seit 1979 wird BoNT zur Behandlung am Menschen angewendet, zunächst zur Therapie der Schielerkrankungen und des Blepharospasmus. Inzwischen wird diese Substanz als ein etabliertes Medikament zur Behandlung verschiedener Erkrankungen wie Torticollis spasticus, Achalasie, Analfisuren, Frey-Syndrom und Sialorrhoe (Amyotrophe Lateralsklerose (ALS), Morbus Parkinson, Zerebralparese) u.a. eingesetzt, vor allem zunehmend in der Neurologie, Viszeralchirurgie und Hals-Nasen-Ohren-Heilkunde. Vorteile liegen in seinem lokalen Wirkungs- und geringen Nebenwirkungsspekrum. Systemische Nebenwirkungen sind in dem angewendeten Dosisbereich nicht zu erwarten [19,20].

Tierexperimentell wurde bereits 1923 über die sekretionshemmende Wirkung von BoNT durch Reduktion des stimulierten Speichelflusses bei Katzen berichtet. Die intraglanduläre Applikation von BoNT führt zur pharmakologischen Denervierung der Speicheldrüsen, in dem es die Acetylcholinausschüttung an der neuroglandulären Synapse inhibiert (Parasympathektomie). Dies führt zu einer Reduktion des Speichelflusses. Indirekte Hinweise über den Wirkmechanismus wurden über die Bestimmung der Acetylcholinesterase-Konzentration im Speichel gewonnen. Ein geringeres Angebot des Transmitters, durch Hemmung seiner Ausschüttung in den synaptischen Spalt durch BoNT, führt zu einer Herabregulation der Acetylcholinesterase, welche für Inhibierung des Acetylcholins zuständig ist. Eine Reduktion der Acetylcholinesterase wurde durch Injektion von BoNT A in die Glandula parotis nachgewiesen [21].

9.3 Hinweise für Handhabung von Botulinum-Toxin

Keine spezielle Handhabung notwendig.

9.4 Relevante Nebenwirkungen im Zusammenhang mit Botulinum-Toxin

In der geplanten Dosierung und bei der Anwendung im Bereich der Speicheldrüsen sind keine relevanten Nebenwirkungen zu erwarten. Die Nebenwirkungen, die in den Fachinformationen (s. Prüfarztordner) dargestellt sind, beziehen sich insbesondere auf die intramuskuläre Anwendung. In dieser Untersuchung wird das Medikament intraglandulär appliziert. Dennoch können folgende in der Fachinformation genannte allgemeine Nebenwirkungen nicht vollends ausgeschlossen werden.

Allgemein

<u>BoNT/A</u>

Wie bei Injektionen zu erwarten, können lokale Schmerzen, Druckempfindlichkeit und/oder Blutergüsse im Bereich der Injektionsstelle auftreten. Nach Injektionen mit Botulinum-Toxin wurden auch Fieber und grippeähnliche Symptome berichtet. Mit der Injektionsnadel in Zusammenhang stehende Schmerzen und/oder Angstgefühle können vasovagale Reaktionen auslösen.

Nebenwirkungen, die mit der Verteilung des Toxins an vom Applikationsort entfernte Stellen in Zusammenhang stehen, wurden sehr selten (<1/10.000) berichtet (übersteigerte Muskelschwäche, Dysphagie, Aspiration/Aspirationspneumonie, in einigen Fällen mit tödlichem Ausgang). Selten (>1/10.000, <1/1.000) wurden schwerwiegende und/oder sofortige Überempfindlichkeitsreaktionen einschließlich Anaphylaxie, Serumkrankheit, Urtikaria, Weichteilödem und Dyspnoe berichtet.

<u>BoNT-B</u>

Die beiden am häufigsten berichteten Nebenwirkungen in einer vergleichenden klinischen Studie der Phase IV bei mit NeuroBloc@10.000 – 15.000 E behandelten Botulinum Toxin-naiven Patienten waren Mundtrockenheit bzw. Dysphagie, die mit einer Häufigkeit von 44 % bzw. 35 % berichtet wurden.

Die aktuellen Fachinformationen für BoNT/A (Botox; Allergan) und BoNT/B (NeuroBloc) sind Bestandteil des Prüfarztordners.

9.5 Relevante Nebenwirkungen im Zusammenhang mit der Behandlung

Die Behandlung wird in der Regel gut und ohne wesentliche Nebenwirkungen vertragen. Dennoch können in seltenen Fällen folgende Nebenerscheinungen auftreten: blauer Fleck oder örtliche Blutung im Bereich der Injektion (vor allem bei Vorbehandlung mit gerinnungshemmenden Medikamenten wie Aspirin), örtliche Infektion und Allergie. Systemische Nebenwirkungen sind in dem angewendeten Dosisbereich nicht zu erwarten.

9.6 Hersteller und Kennzeichnung der Studienmedikation

Die in der geplanten Untersuchung eingesetzten Medikamente BoNT/A (Botox; Allergan, Westport, Ireland) und BoNT/B (NeuroBloc; Eisai Manufactoring, Hertfordshire, UK) werden über die Apotheke der hiesigen Universitätsklinik bestellt.
Der zuständige Apotheker erhält vom KKS eine Liste, die einer bestimmten KKS-internen Nummer eine spezifische Behandlung (1-4) zuteilt. Entsprechend dieser Zuteilung bereitet er nach Mitteilung über Randomisation eines Patienten durch das KKS die Studienmedikation pro KKS-internen Nummer zu und kennzeichnet diese entsprechend den Vorgaben nach § 5 Abs.3+5 GCP-V.

In einem Medikations-log (drug-accountability) muss er genaue Aufzeichnung über die Ausgabe und verwendete Charge pro eindeutiger KKS-internen Nummer führen. Der Monitor wird diesen Log, der in der Apotheke in einem studienseparaten Ordner aufzubewahren ist, bei den vorgesehenen Monitoring-Besuchen überprüfen. Die verbrauchte Studienware (Ampullen, Umverpackung inkl. den Labels) ist vom Prüfer an die Apotheke zurückzugeben.

Zu dokumentieren sind:

- Patienten-Identifikationsnr.
- Eindeutige KKS-interne Nummer
- Gruppenzuteilung (1-4)
- Datum der Ausgabe
- Charge
- Menge
- Verfallsdatum
- Injektionsseite
- Entsorgung (Datum und Unterschrift)

9.7 Vergleichsmedikation

Eine Applikation von NaCl in gleicher Weise (Volumen, Ampullengröße, Aussehen) erfolgt in die gegenseitige, nicht mit BoNT behandelte Gl. submandubularis als Kontrolle zur späteren intraindividuellen Vergleichbarkeit beider Drüsen. Auch diese wird im Zuge der Verblindung entsprechend o.g. Verfahren und den rechtlichen Vorgaben gemäß § 5 Abs. 5 GCP-V. gekennzeichnet.

9.8 Verpackung und Labelling der Studienmedikation und Vergleichsmedikation

Die Umverpackung und die einzelnen Injektionen pro Patient pro Applikation werden gmäß §5 (3+5) GCP-V gekenntzeichnet.
Da es sich um eine verblindete Studie handelt, wird auf Bezeichnung/Stärke des Prüfpräparates, Chargenbezeichnung und Verfallsdatum verzichtet. Stattdessen wird die eindeutige KKS-interne Nummer sowohl auf dem Umkarton als auch auf den Einzelinjektionen vermerkt. Über diese Nummer ist eine eindeutige Zuordung der Studienmedikation anhand der Dokumentation des Apothekers (s.oben) möglich und jederzeit nachvollziehbar und bei Bedarf überprüfbar.
Nach Applikation der entsprechenden Injektion muss der Prüfarzt mit seiner Unterschrift und Datum die Applikation auf dem angebrachten Label bestätigen. Dieses Label muss nach Rückgabe der verbrauchten Studienware im studienspezifischen Apothekenordner aufbewahrt werden. Die Vernichtung verbrauchter Studienware und Verpackungen ist vom zuständigen Apotheker auf dem Medikations-log (drug-accountability) mit Unterschrift und Datum zu dokumentieren.

10 Studiendurchführung

Nach erfolgter ausführlicher Information des Patienten, vorliegender Einwilligung des Patienten zur Studienteilnahme und Überprüfung der Ein- und Ausschlusskriterien, werden die nachfolgenden Untersuchungen und Erhebungen im Rahmen dieser Studie durchgeführt. Sämtliche Untersuchungen, die im Rahmen dieser Studie durchgeführt werden müssen, sind in der Studienübersicht (Flow-Chart) zusammengestellt.

10.1 Basisuntersuchungen vor Therapiebeginn (Screening)

Speicheldrüsenfunktionsprüfung

Die Funktionsprüfung der Gl. submandibularis beidseits erfolgt durch eine Speicheldrüsenszintigraphie. Diese Methode gilt seit Jahren als ein etabliertes Verfahren und stellt die Methode der Wahl zur Funktionsprüfung der Speicheldrüsen dar (s. Anhang 1). Im Rahmen der szintigraphischen Untersuchung wird bei jedem Patienten unter anderem die prozentuale Aufnahme (Uptake) des intravenös applizierten Radionuklids durch die rechte bzw. linke Gl. submandibularis sowie die Ejektionsfraktion nach Gabe eines Reizgetränks (Zitronensaft) bestimmt. Beim prozentualen Uptake handelt es sich um die Größe, welche die Speicheldrüsenfunktion am besten wiedergibt; aus diesem Grund wird das Hauptzielkriterium auf der Basis des prozentualen Uptakes definiert (siehe Abschnitt 15, Statistische Methoden).

10.2 Untersuchungen während der Behandlungsphase

keine!

10.3 Studientherapie

__**Intraglanduläre Injektion von Botulinum-Toxin**__

BoNT soll vor der geplanten Strahlentherapie je nach Zuteilungsergebnis in die rechte oder linke Gl. submandibularis appliziert werden. Applikation von gleichem Volumen NaCl erfolgt in die gegenseitige Gl. submandubularis, die als Kontrolldrüse der späteren intraindividuellen Vergleichbarkeit dient.

Das Medikament soll wie zur Behandlung anderer Speichedrüsenerkrankungen, wie es z.B. bei Hypersalivation üblich und etabliert ist, eingesetzt werden. Es wird sonographisch gesteuert (Sonoline G60 S, Siemens, Erlangen; 7,5-MHz-Linear-Schallkopf) transkutan in das Parenchym der Gl. submandibularis mit einer 27-G-Kanüle appliziert werden.

Bei sechs zufällig ausgewählten Patienten soll die Injektion von BoNT/A in einer Dosierung von 15 U erfolgen. In den sechs anderen Patienten ist die Injektion mit einer Kombination von BoNT/A in einer Dosierung von 15 U und BoNT/B in einer Dosierung von 750 U geplant. Diese Angaben basieren zum einen auf den bisher erzielten Ergebnissen in Vorversuchen (siehe oben) und zum anderen auf Behandlung von Patienten mit Hypersalivation [22,23]. Basierend auf den Ergebnissen der Vorversuche (siehe oben) und bisherigen Daten aus der Literatur zur Therapie von mit Hypersalivationstörungen einhergehenden Krankheitsbildern

ist die Speichelflussrate nach 2 Wochen auf Minimum reduziert [24], so dass die Medikamentenapplikation in der geplanten Studie 2 Wochen vor dem Bestrahlungsbeginn erfolgen soll. Die Strahlentherapie beträgt etwa 7 Wochen, die Funktionsprüfung der Gl. Submandibularis beidseits erfolgt anschließend durch Speicheldrüsenszintigraphie etwa 4 Monate nach Beendigung der Strahlentherapie.

10.4 Begleitmedikation und begleitende Strahlen- und Chemotherapie

Strahlen- und Chemotherapie

Sowohl die Strahlen als auch die Chemotherapie soll nach dem aufgeführten Standard-Protokoll der hiesigen Klinik für Strahlentherapie erfolgen (Zielvolumen: 50,4 Gy, Boost: 9 Gy) (s. Anhang 1).

Supportive Maßnahmen

Während des gesamten Therapieverlaufs ist es seitens der untersuchenden Ärzte zulässig, alle supportiven Medikamente und Behandlungen zu verschreiben, die einer angemessenen Krankenversorgung entsprechen. Diese als auch jede begleitende Medikation 2 Wochen vor Studienbeginn bis zum Ende der jeweiligen Studientherapie ist in dem entsprechenden Dokumentationsbögen (CRF) des Patienten zu erfassen.

11 Untersuchungsmethoden

Speicheldrüsenszintigraphie (bitte siehe 10.1)

12 Sicherheit

12.1 Definitionen zu unerwünschten Ereignissen

Unerwünschtes Ereignis (AE Adverse Event)

Ein Adverse Event (AE) ist definiert als jedes unerwünschte medizinische Ereignis, das bei einem Patient oder bei einem Teilnehmer an einer klinischen Prüfung nach Verabreichung eines Arzneimittels auftritt, und das nicht unbedingt in ursächlichem Zusammenhang mit dieser Behandlung steht. Ein Adverse Event (AE) kann daher jede ungünstige und unbeabsichtigte Reaktion (einschließlich eines anomalen Laborbefundes), jedes Symptom oder jede vorübergehend mit der Verabreichung eines Arzneimittels (hier: eines Prüf-

präparates) einhergehende Erkrankung sein, ob diese nun mit dem Prüfpräparat in Zusammenhang stehen oder nicht. (§ 3 (6) GCP-V).

Nebenwirkung (AR Adverse Drug Reaction)
ist jede nachteilige und unbeabsichtigte Reaktion auf ein Prüfpräparat, unabhängig von dessen Dosierung (§ 3 (7) GCP-V).

Schwerwiegendes unerwünschtes Ereignis (SAE Serious Adverse Event) oder schwerwiegende Nebenwirkung (SAR Serious Adverse Reaction); §3 (8) GCP-V
ist jedes unerwünschte Ereignis, das

- tödlich oder lebensbedrohend ist
- eine stationäre Behandlung oder deren Verlängerung erforderlich macht
- zu bleibender oder schwerwiegender Behinderung oder Invalidität führt
- eine kongenitale Anomalie oder einen Geburtsfehler zur Folge hat.

Der Begriff „lebensbedrohlich“ bezieht sich auf ein Ereignis oder eine Nebenwirkung, das oder die für den Proband zum Zeitpunkt der Reaktion ein tödliches Risiko ausmacht. Er bezieht sich nicht auf eine Reaktion, die hypothetisch zum Tode führen könnte, wenn sie sich zu einem höheren Schweregrad fortentwickelt und u.U. zu Komplikationen geführt hätte.
Danach wird die Schwere eines Ereignisses oder einer Nebenwirkung weitgehend durch die Folge des Krankheitsgeschehens bestimmt. Bei Zweifeln bezüglich des Erfordernisses einer stationären Behandlung sollte die Einstufung der Nebenwirkung als „schwerwiegend“ dann erfolgen, wenn eine stationäre Behandlung des Probanden aufgrund einer aufgetretenen Nebenwirkung tatsächlich stattgefunden hat. In der Regel zählt als stationäre Aufnahme ein Krankenhausaufenthalt, der eine Übernachtung einschließt.

Die Vorstellung eines Patienten in der Notaufnahme allein ohne nachfolgende stationäre Aufnahme erfüllt das Kriterium „Hospitalisierung“ noch nicht. In diesem Zusammenhang ist jedoch zu prüfen, ob ggf. andere der vorgenannten Kriterien die Einstufung der Nebenwirkung als „schwerwiegend“ oder zumindest medizinisch bedeutsam (s.u.) rechtfertigen.

Unerwartete Nebenwirkung (UAR Unexpected Adverse Reaction)
ist eine Nebenwirkung, die nach Art oder Schweregrad nicht mit der vorliegenden Information über das Prüfpräparat übereinstimmt (§ 3 (9) GCP-V).

Verdachtsfall einer unerwarteten schwerwiegenden Nebenwirkung (SUSAR Suspected Unexpected Serious Adverse Reaction)

Jedes schwerwiegende unerwünschte Ereignis, für das entweder der Prüfer und/oder die für die Zweitbewertung verantwortliche Stelle den Kausalzusammenhang als zumindest möglich beurteilt, wird als Verdachtsfall einer schwerwiegenden Nebenwirkung (**S**erious **A**dverse **R**eaction, **SAR**) gewertet.

Sobald dieses Ereignis zusätzlich als unerwartet bewertet wird, handelt es sich um einen Verdachtsfall einer schwerwiegenden unerwarteten Nebenwirkung (**S**uspected **U**nexpected **S**erious **A**dverse **R**eaction, **SUSAR**) (§ 13 (2/3) GCP-V)

12.2 Beurteilung der Intensität

Der Schweregrad eines unerwünschten Ereignisses ermöglicht die qualitative Beurteilung des Ausmaßes bzw. der Stärke eines unerwünschten Ereignisses nach dem Urteil des Prüfarztes bzw. dem Bericht durch den Patient. Der Schweregrad gibt jedoch nicht wieder, ob das Ereignis schwerwiegend ist, sondern nur den Grad bzw. das Ausmaß der Beeinträchtigung bzw. des Ereignisses (z.B. schwere Kopfschmerzen, schwache Müdigkeit). Der Schweregrad sagt auch nichts über den Zusammenhang mit der Medikation bzw. Behandlung aus.

- Grad I: leicht
- Grad II: mittel
- Grad III: schwer

12.3 Beurteilung des Kausalzusammenhangs

Folgende Kategorien eines Zusammenhangs des unerwünschten Ereignisses mit der Gabe der Studienmedikation sind möglich und müssen vom Prüfarzt beurteilt/ eingestuft werden:

1. gesichert
2. wahrscheinlich
3. möglich
4. unwahrscheinlich
5. kein Zusammenhang
6. nicht zu beurteilen

12.4 Dokumentation der Adverse Events (AE)

Sämtliche Adverse Events, die in der Zeit zwischen dem Datum der Einwilligungserklärung und dem Ende der protokollgemäßen Studienvisiten auftreten, müssen im CRF auf der Seite

"Adverse Event" (AE) dokumentiert werden. Grundlage für die Beurteilung des Schwere-grades ist das Urteil des Prüfarztes bzw. der Bericht des Patienten.

12.5 Beobachtung / Weiterbehandlung von Studienteilnehmern mit Adverse Events (AE)

Jedes Adverse Event (AE) muss bis zu dessen Beendigung oder bis zum Zeitpunkt der protokollgemäßen Beendigung der Studienvisiten oder bis zu dem Zeitpunkt, zu dem keine weitere Änderung im outcome mehr erwartet wird, nachbeobachtet werden. Es ist die Aufgabe des Prüfarztes, alle notwendigen Maßnahmen zur Behandlung des Adverse Event (AE) zu ergreifen.
Im Falle des Auftretens von erheblichen unerwünschten Ereignissen werden die Patienten so lange in der Klinik weiter betreut, bis diese vollständig abgeklungen sind.

12.6 Dokumentation von Serious Adverse Events (SAE) / Serious Adverse Drug Reactions (SAR)

Sämtliche Serious Adverse Events / Serious Adverse Drug Reactions, **die in der Zeit zwischen dem Datum der Einwilligungserklärung und 30 Tage nach letzter Verabreichung der Studienmedikation** auftreten, müssen als "Serious Adverse Event“ (SAE) gemeldet und dokumentiert werden.

12.7 Procedere für die unverzügliche Meldung von Serious Adverse Events (SAE)/ Serious Adverse Drug Reactions (SAR) und unerwarteten schwerwiegenden Nebenwirkungen (SUSAR)

12.7.1 Meldung von SAES an den Zweitbewerter/Leiter der klinischen Prüfung

Jedes schwerwiegende unerwünschte Ereignis muss innerhalb von 24h nach dessen bekannt werden vom Prüfarzt an Prof. Dr. J.A. Werner, Direktor der Klinik für HNO-Heilkunde der Philipps-Universität Marburg weitergeleitet werden, auch wenn kein Zusammenhang mit der Studie vermutet wird. Prof. Dr. J.A. Werner führt eine Zweitbewertung des Ereignisses durch.
Sollte die Erstmeldung eines schwerwiegenden unerwünschten Ereignisses per Telefon erfolgen, muss unverzüglich eine schriftliche Meldung folgen.
Dafür muss der Prüfarzt den Bogen zur Dokumentation von schwerwiegenden unerwünschten Ereignissen (SAE) ausfüllen und den Zusammenhang dieser Ereignisses mit

der Studienmedikation beurteilen. Dieses vervollständigte SAE-Formular wird an Prof. Dr. J.A. Werner zur Zweitbewertung weitergeleitet.

12.7.2 Zweitbewertung der Serious Adverse Events(SAE) / Serious Adverse Drug Reactions (SAR)

Die medizinisch-inhaltliche Zweitbewertung der vom Prüfarzt gemeldeten SAEs / SARs erfolgt unverzüglich durch Prof. Dr. J.A. Werner. Es werden nachstehende Punkte bewertet:

1. Zusammenhang zur Medikation ? 1 = Zusammenhang 2 = kein Zusammenhang

2. unerwartetes Ereignis? 1 = unerwartet 2 = erwartet
(unerwartet laut aktuellen Fachinformationen)

3. Beeinträchtigung der Sicherheit / erneute Nutzen-Risiko-Analyse notwendig?
(falls „ja" ist eine schriftliche Nutzen-Risiko-Bewertung durchzuführen)

4. Maßnahmen zum Schutz der betroffenen Personen notwendig?

Falls 1. Zusammenhang und 2. unerwartet ergeben: ➔ SUSAR (siehe nachfolgend!)

Sofern weitere Informationen zur Klärung des schwerwiegenden unerwünschten Ereignisses erforderlich sind, wird der Studienleiter mit dem Prüfarzt in Verbindung treten um weitere, für eine Zweitbewertung nötige, Informationen ein zu holen.

Das SAE inklusive der Zweitbewertung muss Prof. Dr. J.A. Werner spätestens einen Werktag nach Erhalt des SAEs an das KKS Marburg faxen:
Fax: +49 (0) 6421 286 6517

12.8 Ausnahmeregelung bei der Meldung von Serious Adverse Events (SAE)/ Serious Adverse Drug Reactions (SAR)

Die nachfolgenden Ereignisse sind von der Meldepflicht ausgeschlossen:
- Tod als Folge der Tumorprogression

- Stationäre Aufnahmen, bedingt durch chirurgische Eingriffe oder sonstige routinemäßige Behandlungsmaßnahmen von Erkrankungen, die schon vor Studienbeginn bestanden haben und sich während der klinischen Prüfung **nicht verschlechtert haben**.
- Stationäre Aufnahmen, die sich aus den Vorgaben des Studienprotokolls begründen. Hierzu zählen insbesondere: - Applikation der Studienmedikation
  - Kontrolluntersuchungen / Diagnostik

12.8.1 Meldung von SUSARS

Ein SUSAR muss unverzüglich, spätestens aber innerhalb von 15 Tagen nach dem Bekanntwerden

- an die federführende Ethikkommission (Philipps-Universität Marburg)
- die zuständige Bundesoberbehörde (BfArM)
- an die an der klinischen Prüfung beteiligten Prüfer (Information und Ablage im Prüfarzt-Ordner) weitergeleitet werden (§ 13 (2) GCP-V).

Bei einem Verdachtsfall einer unerwarteten schwerwiegenden Nebenwirkung, die zu einem Todesfall geführt hat oder lebensbedrohlich ist, müssen alle für die Bewertung wichtigen Informationen unverzüglich, spätestens aber innerhalb von sieben Tagen an die genannten Stellen übermittelt werden und innerhalb von weiteren acht Tagen die weiteren relevanten Informationen (§ 13 (4) GCP-V).

Die Meldungen von SUSARs an oben genannte Stellen führt das KKS Marburg nach Erhalt der Zweitbewertung durch.

12.8.2 Form der Meldung von SUSARS

Sollte ein SUSAR vorliegen, so erfolgt die Übersendung von Einzelfallberichten über schwerwiegende Nebenwirkungen an die zuständige Bundesoberbehörde mit dem Bogen 643 des BfArM (Bekanntmachung zur Anzeige von Nebenwirkungen und Arzneimittel-missbrauch nach § 63b Abs. 1 bis 8 des Arzneimittelgesetzes (AMG)).
Dieses Formular ist durch die für die Zweitbewertung verantwortliche Person auszufüllen und zusammen mit der SAE-Bewertung unverzüglich an das KKS Marburg zu faxen.

Zusammen mit SAE Form und SAE-Bewertung wird das ausgefüllte BfArM-Formular durch das KKS Marburg an o. g. Stellen gesendet.

12.8.3 Beobachtung von Patienten mit Serious Adverse Event (SAE) und Erstellung eines SAE-Follow-Up

Jedes SAE / SAR ist bis zum Zeitpunkt der Beendigung zu beobachten. Es ist die Aufgabe des Prüfarztes, alle Maßnahmen zur Behandlung des SAE/SAR zu ergreifen. Gegebenenfalls ist ein "SAE-Follow-Up" (Bericht über Behandlung und Ausgang des SAE/SAR) zu erstellen. Das SAE-Follow-Up wird im CRF auf dem Formular "Serious Adverse Event (SAE) dokumentiert und genauso gehandhabt wie das ursprüngliche SAE (s.12.7)

12.9 Weitere ggf. erforderliche Meldungen

Falls Überprüfung der Nutzen-Risiko-Bewertung erforderlich (§13 (4) GCP-V)

Hat sich bei der Bewertung des SAE herausgestellt, dass eine erneute Überprüfung der Nutzen-Risiko-Bewertung der klinischen Prüfung erforderlich ist, sind

- die zuständige Bundesoberbehörde (BfArM)
- die federführende Ethikkommission (Philipps-Universität Marburg)

unverzüglich, spätestens aber innerhalb von 15 Tagen nach Bekanntwerden über den auslösenden Sachverhalt zu unterrichten.

Hierzu gehören insbesondere

- Einzelfallberichte von erwarteten schwerwiegenden Nebenwirkungen mit einem unerwarteten Ausgang,
- eine Erhöhung der Häufigkeit erwarteter schwerwiegender Nebenwirkungen, die als klinisch relevant bewertet wird,
- Verdachtsfälle schwerwiegender unerwarteter Nebenwirkungen, die sich ereigneten, nachdem die betroffene Person die klinische Prüfung bereits beendet hat,
- Ereignisse im Zusammenhang mit der Studiendurchführung oder der Entwicklung des Prüfpräparates, die möglicherweise die Sicherheit der betroffenen Personen beeinträchtigen können.

Die erforderliche Meldung an oben genannte Stellen nimmt nach erneuter schriftlicher Nutzen-Risiko-Bewertung durch den Zweitbewerter das KKS vor.

Falls Maßnahmen zum Schutz vor unmittelbarer Gefahr erforderlich (§13 (5) GCP-V)

Hat sich laut der eingeholten Bewertung des SAE herausgestellt, dass Maßnahmen ergriffen werden müssen zum Schutz der betroffenen Personen vor unmittelbarer Gefahr oder dass neue Umstände die Sicherheit der betroffenen Personen beeinträchtigen können, so sind unverzüglich

- die zuständige Bundesoberbehörde (BfArM)
- die zuständige lokale Behörde (RP Darmstadt)
- die federführende Ethikkommission

über diese Maßnahmen sowie die sie auslösenden Umstände zu unterrichten.

Der Zweitbewerter erstellt diese Meldungen, und das KKS Marburg leitet diese an die oben genannten Stellen weiter.

12.10 Jahresbericht (Annual Safety Report)

Die in §13 (6) GCP-Verordnung geforderte Erstellung des Jahresberichtes (Annual Safety Report), über alle während der Studie aufgetretenen SUSARs und SARs, erfolgt durch den Leiter der klinischen Prüfung in Zusammenarbeit mit dem KKS Marburg Der Jahresbericht wird anschließend vom KKS an

- die zuständige Bundsoberbehörde (BfArM)
- die zuständige Ethikkommission (Philipps-Universität Marburg)

weitergeleitet.

12.11 Begleiterkrankungen

Begleiterkrankungen werden beim Screening anamnestisch erhoben und bis zum Studienende des jeweiligen Patienten im CRF dokumentiert.

12.12 Schwangerschaft

Studienpatientinnen, bei denen im Studienverlauf eine Schwangerschaft diagnostiziert wird, sind **sofort aus der Studie auszuschließen**. Der Prüfarzt ist verpflichtet, den Sponsor unverzüglich nach bekannt werden der Schwangerschaft zu benachrichtigen. Der Prüfarzt sollte den weiteren Verlauf der Schwangerschaft verfolgen und im Rahmen eines Follow-Up-

Reports Informationen über die Geburt/Stillzeit des Kindes zur Verfügung stellen. Hierzu wird, wie für die Initialmeldung, das Formular " Meldung einer Schwangerschaft“ verwendet.

12.13 Überdosierung

Eine Überdosierung bezeichnet die versehentliche oder bewusste Überschreitung der zur Therapie erforderlichen und festgelegten Dosis.
Eine durch den Prüfarzt festgestellte Überdosierung ist unverzüglich nach bekannt werden auf dem Formular "Serious Adverse Event (SAE)" zu dokumentieren und an den Leiter der klinischen Studie zu melden (s10.7). Es ist die Aufgabe des Prüfarztes, falls nötig alle notwendigen Maßnahmen zur Behandlung der Überdosierung zu ergreifen.

12.14 Andere medizinisch besonders bedeutsame Ereignisse

Die Bundesoberbehörden empfehlen darüber hinaus, medizinisch bedeutsame Nebenwirkungen als „schwerwiegend“ einzustufen. Das sind solche, die nicht sofort tödlich oder lebensbedrohlich sind oder zu einer stationären Behandlung führen müssen, aber den Patienten erheblich beeinträchtigen können. Medizinisch bedeutsam sind Nebenwirkungen auch dann, wenn sie eine Intervention/Behandlung zur Verhinderung eines Zustandes erfordern, der den in der Definition „schwerwiegende Nebenwirkung“ genannten Kriterien entspricht (vgl. BfArM, 2005: 8).

13 Administrative Aspekte

13.1 Dokumentation der Daten im CRF

Das Prüfzentrum hat für jeden Patienten einen Erhebungsbogen (Case Report Form = CRF). Die im Rahmen der Studie erhobenen Patientendaten werden ohne Nennung des Namens in das CRF eingetragen und der Patient ist nur durch eine Zentrums- und Patientennummer (Identifikationsnummer) gekennzeichnet. Falls, in Ausnahmefällen, aus Sicherheitsgründen oder aufgrund gesetzlicher Vorschriften die Kenntnis der Identität des Patienten erforderlich wird, wird diese sowohl vom Studienleiter als auch vom Prüfarzt vertraulich behandelt. Die Eintragungen in das CRF müssen deutlich mit schwarzem Kugelschreiber gemacht werden, um die Leserlichkeit auf Durchschreibesätzen oder auf kopierten Seiten sicherzustellen.
Alle vom Prüfplan geforderten Daten sollen zur Verfügung gestellt werden. Das Fehlen von Daten muss begründet werden:
| ND| = not done (nicht durchgeführt)

| NA |= not applicable (nicht zutreffend)

| NK | = not known (nicht bekannt)

Korrekturen werden vorgenommen, indem die falsche Eintragung mit einer einzigen horizontalen Linie durchgestrichen wird, so dass sie leserlich bleibt und die neue Eintragung wird daneben geschrieben. Die neue Eintragung muss von einem für Eintragungen in das CRF autorisierten Mitglied des Studienteams mit Initialen und Datum abgezeichnet werden:

2 5 1 1 0 2

| ~~1 | | 3 | | 0 | | 5 | | 0 | | 3~~ | TM 26.06.05

TT MM JJ

13.2 Originaldaten und Originaldokumente

Der Prüfarzt muss für jeden Studienpatienten Original-Dokumente führen. Jede Information im CRF muss auf diese Original-Dokumente zurückgeführt werden können, die im Allgemeinen in der Patienten-Krankenakte aufbewahrt werden. Die Original-Dokumente müssen alle demographischen und medizinischen Informationen, klinische Befunde, Labordaten, Arztberichte, Röntgenbilder, Aufzeichnungen zur Applikation von Arzneimitteln/Studienmedikation, Informationen zu AEs und SAEs/SARs etc. enthalten. Eine Kopie der unterschriebenen Einverständniserklärung, auf der der Titel der Studie vermerkt sein muss, ist der Krankenakte beizufügen. Anhand der Originaldaten sollte es möglich sein, die Ein- und Ausschlusskriterien des betreffenden Studienpatienten zu überprüfen.

13.3 Sicherstellung der Datenqualität, Monitoring, Audit

Monitoring

Das Monitoring erfolgt durch das KKS vor, 1x während und nach dem Ende der Studie gemäß den entsprechenden SOPs des KKS Marburg.

Das Monitoring dient dazu, die Eintragungen im Dokumentationsbogen (Case Report Form = CRF) auf Richtigkeit und Vollständigkeit und, soweit Vorhanden, im Vergleich zu Original-daten, zu überprüfen und somit die Qualität der Daten zu sichern und zu erhöhen. Zur Durchführung des Monitorings gewährt der Prüfarzt und er Apotheker dem Monitor Einsicht in die CRF-Ordner der Patienten, die Krankenakten der Ptienten und in sonstige studienspezifischen Akten, wie z.B. den ISF und den studienspezisfischen Apothekenordner, welcher in der Apotheke gelagert und geführt wird .

100%ige Überprüfung

Daten, die bei allen Patienten zu 100% überprüft werden sind:

- Ausgabe und Dokumentation des Prüfpräparats
- Unerwünschte Ereignisse
- Ein-/Ausschlußkriterien
- Einwilligungserklärung

Grundsätzlich werden fehlende oder unplausible Daten im Rahmen des Monitorings mit dem Prüfarzt geklärt und erforderliche Ergänzungen und Korrekturen in den Prüfbogen GCP-gerecht vorgenommen.

Audit / Inspektionen

Diese Studie wird gegebenenfalls von Überwachungsbehörden und/oder im Auftrag des Sponsors von einer unabhängigen Abteilung für Qualitätssicherung überprüft. Dazu gehört, dass die Auditoren/Inspektoren das Recht haben, das Studienzentrum während und/oder nach Ende der Studie zu besuchen und Zugang zu studienbezogenen Dokumenten erhalten. Mit der schriftlichen Bestätigung seiner Teilnahme an dieser Prüfung erklärt sich der Hauptprüfer mit diesen Anforderungen einverstanden.

Die Studienleitung und der Sponsor versichern, dass zum Schutz des Patienten alle Daten, die der Prüfarzt an die Studienleitung und den Sponsor weitergibt, jederzeit vertraulich behandelt werden.

13.4 Prüfarztordner (Investigator Site File) / Archivierung des Prüfarztordners

Vor Beginn der Studie im Prüfzentrum, wird im KKS ein Prüfarztordner (ISF) erstellt. Dieser enthält alle essentiellen Unterlagen zur Durchführung der klinischen Prüfung und alle Dokumente, die während des Verlaufs der klinischen Prüfung beim Hauptprüfer eingehen, erstellt und ergänzt werden. Unter anderem enthält er die Notfallumschläge aller Patienten, die mit der eindeutigen KKS-internen Nummer gekennzeichnet sind.

Der Hauptprüfer am Prüfzentrum ist für die Archivierung des Prüfarztordners verantwortlich. Die Archivierungszeit beträgt mindestens 10 Jahre nach Abschluss der Studie.

13.5 Datenmanagement und Archivierung

Die Dateneingabe und die Erstellung von Queries erfolgt im Koordinierungszentrum für Klinische Studien Marburg, die Datenerfassung ggf. auch durch einen Doktoranten von Prof. Teymoortash. Die Auswertung erfolgt durch Prof. Strauch (Institut für Medizinische Biometrie und Epidemiologie der Universität Marburg).. Die Archivierung der studienrelevanten Daten sowie die auf elektronischen Datenträgern gespeicherten Ergebnisse werden mind. 10 Jahre

nach Abschluss dieser Studie im Prüfzentrum aufbewahrt, wofür der Leiter der Klinischen Prüfung die Verantwortung trägt.

13.6 Datenschutz

Gemäß § 40 Abs. 2 AMG werden sämtliche patientenbezogenen medizinischen Daten nur in pseudonymisierter Form zusammen mit einer 4-stellingen Identifikationsnummer gespeichert, nicht aber mit Name oder Adresse des Patienten. Sämtliche Daten, die elektronisch gespeichert werden, sind durch ein Passwort geschützt und werden ausschließlich verschlüsselt übertragen.

14 Ethische und rechtliche Aspekte

14.1 Verantwortlichkeit des Hauptprüfers (Investigator) am Prüfzentrum

Der Hauptprüfer am Prüfzentrum ist verantwortlich, dass die vorliegende klinische Prüfung in Übereinstimmung mit der Deklaration von Helsinki in der letzten allgemein anerkannten gültigen Fassung (1996 Sommerset West, Republic of South Africa, Anlage B), der „ICH Guideline of Good Clinical Practice (ICH-GCP, Topic E6, 1995), der GCP-Verordnung (GCP-V / August 2004) und dem deutschen Arzneimittelgesetz (AMG 12. Novelle / August 2004 - §40 und §41) durchgeführt wird.

14.2 Patienteninformation und Patienteneinwilligung

Der Prüfarzt muss jeden Studienpatienten über Wesen und Art der Studie, ihr Ziel, die durchgeführten Untersuchungen, die geplante Dauer, mögliche Risiken und Nutzen und jegliche Unannehmlichkeiten, die damit verbunden sein könnten, aufklären. Jeder Studienpatient muss darüber informiert werden, dass die Teilnahme an der Studie freiwillig ist, und dass er jederzeit aus der Studie ausscheiden kann. Informationen zum Datenschutz werden dem Patienten ebenso erläutert, wie die Bestimmungen der Patientenversicherung. Der Patient muss die Möglichkeit haben, Fragen zu stellen und erhält nach der mündlichen Aufklärung eine Kopie der schriftlichen Patienteninformation.

Das Einverständnis zur Teilnahme an der klinischen Prüfung muss mit Hilfe einer standardisierten schriftlichen Erklärung in für Laien verständlicher Sprache vor dem Screening eingeholt werden. Der Patient muss die Erklärung zur Einwilligung lesen und überdenken, bevor er sie signiert und datiert. Eine Kopie des unterschriebenen Dokuments muss dem Patienten ausgehändigt werden. Es kann kein Patient in die Studie aufgenommen werden, der nicht zuvor die Einverständniserklärung unterschrieben hat.

Aufklärung und Einholen der Einwilligung ist eine ärztliche Tätigkeit und ausschließlich Prüfärzten vorbehalten.

14.3 Patientenversicherung

Vor Beginn einer klinischen Prüfung muss gemäß Arzneimittelgesetz § 40 Abs. 1 und Abs. 3 und ICH-GCP Punkt 5.8 eine bestehende Patientenversicherung nachgewiesen werden. Die Patientenversicherung dient der finanziellen Absicherung von Gesundheitsschäden von Studienteilnehmern, die in unmittelbarem Zusammenhang mit der klinischen Prüfung stehen.
Für jeden Patienten ist eine entsprechende Versicherung bei der Gerling Vertrieb Deutschland GmbH abgeschlossen worden. Die Patienten werden durch den Prüfarzt über den Abschluss und den Inhalt der Patientenversicherung informiert und erhalten eine Kopie der Versicherungsunterlagen und der Allgemeinen Versicherungsbedingungen.
Adresse der Versicherung:

Gerling Industrie Versicherung AG
Vertragssevice Haftpflicht
Am Schönenkamp 45
D-40599 Düsseldorf
Tel.: 0211-7482 5404
Fax: 0211-7482 565

14.4 Bewertung durch die Ethikkommission nach AMG

Nach § 40 Abs. 1 des Arzneimittelgesetzes (AMG 12; Stand August 2004) darf die klinische Prüfung eines Arzneimittels bei Menschen vom Sponsor nur begonnen werden, wenn die zuständige Ethikkommission diese nach Maßgabe des § 42 Abs. 1 zustimmend bewertet hat. Die Antragstellung auf zustimmende Bewertung der klinischen Prüfung regelt § 7 Abs. 1, 2 und Abs. 3 der GCP-Verordnung (GCP-V).
Die erforderliche zustimmende Bewertung der Ethikkommission ist vom Sponsor bei der für den Prüfer zuständigen Ethikkommission zu beantragen (§ 42 Abs. 1 AMG).
Im Verlauf der klinischen Prüfung wird der Ethikkommission jedes Protokoll-Amendment vorgelegt. Die Unterrichtung über die in der klinischen Prüfung aufgetretenen SUSARs erfolgt nach den gesetzlichen Bestimmungen und den behördlichen Auflagen.

14.5 Behördliche Genehmigungs- und Anzeigeverfahren nach AMG

Nach §40 Abs. 1 AMG, §42 Abs. 2 AMG, §7 Abs. 1, 2 und 4 GCP-Verordnung (GCP-V) und der 3. Bekanntmachung zur klinischen Prüfung von Arzneimitteln am Menschen (vom 10. August 2006), sind klinische Prüfungen mit Arzneimitteln vor Beginn von der zuständigen Bundesoberbehörde (BOB) zu genehmigen und nach §67 Abs. 1 AMG und §12 Abs.1 GCP-V den zuständigen lokalen Überwachungsbehörden anzuzeigen.
Gemäß §67 Abs. 1 und Abs. 3 AMG sind Sponsoren sowie Einrichtungen, die in deren Auftrag klinische Prüfungen mit Arzneimitteln durchführen, und die verantwortlichen Prüfer in jedem Prüfzentrum verpflichtet, den Beginn, das Ende, den Abbruch, die Unterbrechung und Änderungen einer klinischen Prüfung bei den zuständigen lokalen Überwachungsbehörden und ggf. auch bei der Bundesoberbehörde anzuzeigen.

14.6 Protokoll-Amendment

Jede Veränderung des vorliegenden Studienprotokolls erfordert die Erstellung eines schriftlichen Amendments. Der Leiter der klinischen Prüfung, der Sponsor und der verantwortliche Biometriker müssen dem Amendment zustimmen und das Amendment unterschreiben.

Falls die im Amendment festgelegten Änderungen des Prüfplans

- sich auf die Sicherheit der betroffenen Personen auswirken,
- die Auslegung der wissenschaftlichen Dokumente, auf die die Prüfung gestützt wird, oder die wissenschaftliche Aussagekraft der Studienergebnisse beeinflussen,
- die Art der Leitung oder der Durchführung wesentlich verändern,
- die Qualität oder Unbedenklichkeit der Prüfpräparate beeinträchtigen oder
- bei klinischen Prüfungen mit Arzneimitteln, die aus gentechnisch veränderten Organismen bestehen oder diese enthalten, die Risikobewertung für die Gesundheit nicht betroffener Personen und die Umwelt verändern,

muss das Amendment der zuständigen Bundesoberbehörde und Ethikkommission vorgelegt werden. Erst nach erfolgter Genehmigung und zustimmender Bewertung erhält das Amendment seine Gültigkeit.

14.7 Notwendige Studiendokumente vor Studienbeginn

Bevor das Prüfzentrum mit der Studie beginnen kann, müssen folgende Dokumente vorliegen:

- Zustimmende Bewertung der zuständigen/federführenden Ethikkommission

- Genehmigung der zuständigen Bundesoberbehörde (BOB)
- Anzeige des Beginns der klinischen Prüfung bei der zuständigen BOB
- Anzeige des Prüfzentrums und des Hauptprüfers (Investigator) am Prüfzentrum an die zuständige lokale Behörde (gemäß § 67 AMG)
- Unterzeichneter Prüfarztvertrag
- Das vom Hauptprüfer (Investigator) und den Co-Investigatoren am Prüfzentrum unterzeichnete Studienprotokoll

15 Statistische Methoden

Sowohl vor als auch 4 Monate nach der Behandlung wird für jeden Patienten die Differenz zwischen der szintigraphisch bestimmten prozentualen Aufnahme (Uptake) des applizierten Radionuklids durch die mit BoNT behandelte Drüse (je nach Randomisierungsergebnis rechts oder links) und die gegenseitige Drüse betrachtet. Dadurch wird der Funktionsunterschied zwischen beiden Drüsen gemessen und gleichzeitig eine *intraindividuelle situationsabhängige Variabilität*, die beide Drüsen in gleichem Maße betrifft, so weit wie möglich ausgeschaltet. Das Hauptzielkriterium ist der Unterschied dieser beiden Seitendifferenzen vor und 4 Monate nach der Behandlung, d.h. die Uptake-Differenz behandelte-unbehandelte Drüse 4 Monate nach der Behandlung minus der Differenz behandelte-unbehandelte Drüse vor der Behandlung (Baseline). Damit wird die Änderung des Funktionsunterschiedes zwischen behandelter und unbehandelter Drüse im Verlauf der Behandlung gemessen. Durch den Bezug auf die Baseline läßt sich die *interindividuelle Variabilität*, vor allem bedingt durch unterschiedliche Beschaffenheiten der beiden Drüsen vor Beginn der Behandlung, reduzieren. Mit dem Wilcoxon-Vorzeichen-Rangtest (Wilcoxon signed rank test), welcher keine normalverteilten Werte voraussetzt, wird geprüft, ob innerhalb jeder der beiden Dosisgruppen (BoNT/A sowie BoNT/A und –B, je 6 Patienten) das Hauptzielkriterium signifikant von null abweicht. Ein Erwartungswert null entspricht dabei keinem Behandlungseffekt von BoNT bzw. demselben Effekt von BoNT und Placebo (Nullhypothese). Weiterhin soll mit dem Mann-Whitney-U-Test, der ebenfalls keine normalverteilten Werte voraussetzt, geprüft werden, ob sich die Behandlungseffekte in beiden Dosisgruppen unterscheiden, ebenfalls in bezug auf das Hauptzielkriterium, d.h. den Vorher-Nachher-Unterschied der Uptake-Seitendifferenz. Da es sich um eine Phase-I-Studie handelt, werden p-Werte < 0.05 als nominal signifikant interpretiert. Aufgrund der Ergebnisse bei den Vorversuchen an Ratten wird von einem mittleren Effekt zwischen behandelter und unbehandelter Drüse in zweifacher Höhe der Standardabweichung ausgegangen. Bei diesem Effekt erreicht der Wilcoxon-Vorzeichen-Rangtest unter Annahme normalverteilter

Werte mit 6 Patienten bei einem Fehler 1. Art von 0,05 eine Power von 87% (die Berechnung erfolgte mittels der Statistiksoftware R [25].

Als Nebenzielkriterium soll die Ejektionsfraktion nach Gabe eines Reizgetränks (Zitronensaft) betrachtet werden, und zwar wiederum in Form des Vorher-Nachher-Unterschiedes der Seitendifferenz und unter Anwendung des Wilcoxon-Vorzeichen-Rangtests bzw. des Mann-Whitney-U-Tests (siehe oben). Die Sicherheit und Verträglichkeit der Behandlung mit BoNT werden deskriptiv ausgewertet.

16 Verantwortlichkeit

Der Prüfarzt ist für die Behandlung der Patienten, für die Meldung von SAEs an das KKS und die Dokumentation der CRFs verantwortlich.

Der Studienleiter kann bei medizinischen Sachfragen kontaktiert werden, ist für Erstellung des ASR in Zusammenarbeit mit dem Biometriker und die Archivierung der Dokumente verantwortlich.

Für die Zweitbewertung der gemeldeten SAEs, die Weiterleitung der bewerteten SAEs ans KKS sowie die vollständige Dokumentation von SUSARs ist der Direktor der Klinik, Prof. Dr. J.A. Werner, zuständig und verantwortlich. Des Weiteren ist er für eine ggf. erneute Nutzen-Risiko-Bewertung und die Beschreibung von ggf. notwendigen Maßnahmen zum Schutz vor unmittelbarer Gefahr zuständig.

Das Datenmanagement erfolgt im KKS, die Auswertung im IMBE (Prof. Strauch).

Das KKS Marburg ist ebenfalls für die Einholung der Genehmigungen, die Registrierung und für die Weiterleitung der Safety Meldungen an die Bundesoberbehörde und die Ethikkommission zuständig.

17 Literatur

[1] Porter SR, Scully C, Hegarty AM. An update of the etiology and management of xerostomia. Oral Surg Oral Med Oral Pathol Oral Radiol Endod 2004; 97: 28-46.

[2] Bansal M, Mohanti BK, Shah N, Chaudhry R, Bahadur S, Shukla NK. Radiation related morbidities and their impact on quality of life in head and neck cancer patients receiving radical radiotherapy. Qual Life Res 2004; 13: 481-8.

[3] Naresh J, Hadi S, Timothy m, Coulter L. Submandibular salivary gland

transfer prevents radiation-induced xerostomia. Int J Radiat Oncol Biol Phys 2000; 46: 7-11.

[4] Eisbruch A. Reducing xerostomia by IMRT: what may, and may not, be achieved. J Clin Oncol 2007; 25: 4863-4.

[5] Kam MMK, Leung SF, Zee B, et al: Impact of intensity modulated radiotherapy (IMRT) on salivary gland function in early stage nasopharyngeal carcinoma patients: A prospective randomized study. J Clin Oncol 2007; 25: 4873 -4879

[6] Teymoortash A, Sommer F, Mandic R, Schulz S, Bette M, Aumüller G, Werner JA. Intraglandular application of botulinum toxin leads to structural and functional changes in rat acinar cells. Br J Pharmacol 2007: 152; 161-7.

[7] Teymoortash A, Müller F, Juricko J, Bieker M, Mandic R, Librizzi D, Höffken H, Pfestroff A, Werner JA. Botulinum toxin prevents radiotherapy-induced salivary gland damage. Oral Oncol, im Druck

[8] Ellies M, Laskawi R, Rohrbach-Volland S, Arglebe C, Beuche W. Botulinum toxin to reduce saliva flow: selected indications for ultrasound-guided toxin application into salivary glands. Laryngoscope 2002; 112: 82-86.

[9] Ellies M, Gottstein U, Rohrbach-Volland S, Arglebe C, Laskawi R. Reduction of salivary flow with botulinum toxin: extended report on 33 patients with drooling, salivary fistulas, and sialadenitis. Laryngoscope 2004; 114: 1856-1860.

[10] Ellies M, Laskawi R, Gotz W, Arglebe C, Tormahlen G. Immunohistochemical and morphometric investigations of the influence of botulinum toxin on the submandibular gland of the rat. Eur Arch Otorhinolaryngol 1999; 256: 148-152.

[11] Fujita-Yoshigaki J, Dohke Y, Hara-Yokoyama M, Furuyama S, Sugiya H. Presence of a complex containing vesicle-associated membrane protein 2 in rat parotid acinar cells and its disassembly upon activation of cAMP-dependent protein kinase. J Biol Chem 1999; 274: 23642-23646.

[12] Nashida T, Imai A, Shimomura H. Relation of Rab26 to the amylase release from rat parotid acinar cells. Arch Oral Biol 2006; 51: 89-95.

[13] Fujita-Yoshigaki J, Dohke Y, Hara-Yokoyama M, Kamata Y, Kozaki S, Furuyama S, Sugiya H. Vesicle-associated membrane protein 2 is essential for cAMP-regulated exocytosis in rat parotid acinar cells. J Biol Chem 1996; 271: 13130-13134.

[14] Takuma T, Arakawa T, Tajima Y. Interaction of SNARE proteins in rat parotid acinar cells. Arch Oral Biol 2000; 45: 369-375.

[15] Imai A, Nashida T, Yoshie S, Shimomura H. Intracellular localisation of SNARE proteins in rat parotid acinar cells: SNARE complexes on the apical plasma membrane. Arch Oral Biol 2003; 48: 597-604.

[16] Abok K, Brunk U, Jung B, Ericsson J. Morphologic and histochemical studies on the differing radiosensitivity of ductular and acinar cells of the rat submandibular gland. Virchows Arch B Cell Pathol Incl Mol Pathol 1984; 45: 443-60.

[17] Nagler RM. The enigmatic mechanism of irradiation-induced damage to the major salivary glands. Oral Dis 2002; 8: 141-6.

[18] Binz T, Kuranzono H, Wille M, Frevert J, Wernars K, Niemann H. The complete sequence of botulinum neurotoxin type A and comparison with other clostridial neurotoxins. J Biol Chem 1990; 265: 9153-8.

[19] Ellies M, Laskawi R, Tormählen G, Götz W. The effect of local injection of botulinum toxin A on the parotid gland of the rat: an immunohistochemical and morphometric study. J Oral Maxillofac Surg 2000; 58: 1251-6.

[20] Arad-Cohen A, Blitze A. Botulinum toxin treatment for symptomatic Frey's syndrom. Otolaryngol Head Neck Surg 2000; 122: 237-40.

[21] Jongerius PH, Joosten F, Hoogen FJ, Gabreels FJ, Rotteveel JJ. The Treatment of drooling by ultrasound-guided intraglandular injections of botulinum toxin type A into the salivary glands. Laryngoscope 2003; 113: 107-11.

[22] Krause E, Bötzel K, de la Chaux R, Gürkov R. Lokale Botulinum-Toxin-A-Thjerapie bei chronischer Sialorrhoe. HNO 2008; 56 : 941-5.

[23] Jongerius PH, Rotteveel JJ, van Limbeek J, Gabreëls FJ, van Hulst K, van den Hoogen FJ.Botulinum toxin effect on salivary flow rate in children with cerebral palsy. Neurology 2004; 63: 1371-5.

[24] Rohrbach S, Laskawi R. Praktische Anwendung von Botulinumtoxin im Kopf-Hals-Bereich. HNO 2004; 52: 635-41.

[25] [R Development Core Team (2005). R: A language and environment for statistical computing. R Foundation for Statistical Computing, Vienna, Austria. ISBN 3-900051-07-0, URL http://www.R-project.org]).

18 Anhänge

18.1 Anhang 1: Behandlungsprotokolle

Szintigraphie-Protokoll:

Allgemeines:	Tc-99m-Pertechnetat wird in die Speicheldrüsen aktiv über den Na+/K+/2Cl-Kotransport aufgenommen. Damit lassen sich die Parenchymfunktion und die Abflußverhältnisse der großen Kopfspeicheldrüsen qualitativ beurteilen.
Indikationen:	- Abflußbehinderungen mit/ohne Parenchymschädigung<br>- Parenchymschädigungen, Z. n. hoch-dosierter I-131-Therapie, Z. n. Radiatio
Kontraindikationen:	- SCHWANGERSCHAFT<br>- IRENATGABE
Strenge Indikationsstellung:	Wiederholungsuntersuchung < 3 Monate
Prämedikation:	keine
Vorbereitung:	Patient sollte 4 Std. vor der Untersuchung nüchtern sein.
Radiopharmakon:	Tc-99m-Pertechnetat
Applizierte Aktivität :	200 MBq
Applikationsmodus:	i.v. Applikation<br>(Dokumentation von Aktivitätsmenge, Injektionsstelle, Injektionszeitpunkt und Aufnahmezeitpunkt in der Patientenakte)
Untersuchungsgerät:	Einkopf-Gamma-Kamera
Kollimator:	LEHR (Entfernung Kollimator – Patienten minimieren)
Aufnahmeparameter:	Studienname Salivary Scan<br>Serienname Speicheldrüse<br>Isotop Tc-99m-Pertechnetat<br>Matrix 128 x 128<br>Zoom 2<br>Kameravoreinstellung Tc-99m-NMG<br>Detektoren Detektor 1<br>Orientierung Füße zu Gentry<br>Patientenlage Rückenlage<br>Studienbasiertes Setup aus<br>Phasenbild Parameter<br>Phasenbild 1 1,00“ x 60 Einzelbilder<br>Phasenbild2 60,00“ x 44 Einzelbilder

Aufnahmeposition:	- im Liegen, Kopf gut fixiert und vollständig abgebildet (gerade Kopfhaltung!), Jugulum am unteren Bildrand, Nasenspitze auf Kollimatormitte<br>- bequeme Lagerung des Patienten (Knierolle, Armpolster)
Beginn der Akquisition:	- direkt nach der Injektion<br>- 15 Min. p.i. orale Gabe von Reizgetränk – Zitronensaft (mit Hilfe von z. B. biegsamem Strohhalm; Patient soll den Saft schlucken, ohne den Kopf zu bewegen)
Gesamtdauer:	70 Min. (Anamnese, Aufnahmen, Auswertung Befundbesprechung), davon 45 Min. für die Aufnahmen
Bemerkung:	- keine Gabe von Natriumperchlorat!<br>- Achtung bei der Gabe des Reizgetränks, daß der Patient seinen Kopf nicht bewegt
Auswertung:	- ROI über beide Glandulae parotides und beide Glandulae submandibulares (re. und li.)<br>- Erstellung von Zeitaktivitätskurven
Dokumentation:	Zeitaktivitätskurven auf einem Blatt (Farbscala: CTY4), zwei Ausdrucke

Radiochemotherapieprotokoll:

Strahlentherapie							
Woche	1	2	3	4	5	6	7
EHD: 1,8 Gy	IIIII	IIIII	IIIII	IIIII	IIIII	III	
EHD: 1,8 Gy Boost						II	III
Chemotherapie	x			x			x

Chemotherapie	Dosis (m^2)	Patientendosis	Träger	i.v.	Tage
Cisplatin	100 mg absolut	100mg absolut	0,9 NaCl	i.v.1h	1, 22, 43

Supportivtherapie	Dosis	Träger	Zeitpunkt	Bemerkung
0,9%-NaCl	jeweils 1 l		vor/nach DDP	
Kevatril	1 Amp. (3 mg)		vor DDP	
Fortecortin	4 mg		vor DDP	

Prüfplanunterschriften

Ich habe dieses Protokoll gelesen und stimme zu, die Studie in Übereinstimmung mit allen Festlegungen dieses Protokolls sowie ICH-GCP, der Deklaration von Helsinki (Version 1996) und des AMG und der GCP-Verordnung durchzuführen.

Außerdem stimme ich zu, sämtliche Informationen bezüglich dieser Studie geheim zu halten

Bevollmächtigter des Sponsors (KKS Marburg)

Carmen Schade-Brittinger Datum

Leiter der Klinischen Prüfung

Prof. Dr. med. Afshin Teymoortash Datum

Biometriker

K. Strauch 24.07.2009

Prof. Dr. rer. nat. Konstantin Strauch Datum

Prüfplanunterschriften

Ich habe dieses Protokoll gelesen und stimme zu, die Studie in Übereinstimmung mit allen Festlegungen dieses Protokolls sowie ICH-GCP, der Deklaration von Helsinki (Version 1996) und des AMG und der GCP-Verordnung durchzuführen.

Außerdem stimme ich zu, sämtliche Informationen bezüglich dieser Studie geheim zu halten

Bevollmächtigter des Sponsors (KKS Marburg)

i.V. [signature] 27.07.2009

Carmen Schade-Brittinger Datum

Dr. Michael Wittenberg

Leiter der Klinischen Prüfung

Prof. Dr. med. Afshin Teymoortash Datum

Biometriker

[signature] 24.07.2009

Prof. Dr. rer. nat. Konstantin Strauch Datum
