# Supplementary material for: Safety and Efficacy of Botulinum Toxin to Preserve Gland Function after Radiotherapy in Patients with Head and Neck Cancer: A Prospective, Randomized, Placebo-Controlled, Double-Blinded Phase I Clinical Trial
Source: PLoS One. 2016 Mar 18;11(3):e0151316. doi: 10.1371/journal.pone.0151316 (PMC4798778; doi:10.1371/journal.pone.0151316)
Supplement: S2 Text — (PDF) [file pone.0151316.s005.pdf]

KKS Marburg, Karl-von-Frisch-Strasse 4, 35037 Marburg

An die Ethikkommission der Philipps-Universität Marburg

Prof. Dr. G. Richter

Baldingerstrasse

35033 Marburg

Koordinierungszentrum für Klinische Studien
Leitung: Carmen Schade-Brittinger

Susanne Harnisch

Anschrift	KKS Marburg, Karl-von-Frisch-Strasse 4, 35037 Marburg
Telefon	++49-6421-28 66553
Telefax	++49-6421-28 66517
E-Mail	Susanne.harnisch@kks.uni-marburg.de
Internet	www.kks-mr.de
Datum	08.10.2012

AZ 115/09(A)

Funktionsszintigraphische Untersuchungen zur Überprüfung der radioprotektiven Wirksamkeit intraglandulär applizierten Botulinum-Toxins (BoNT) auf die Gl. submandibularis bei Patienten mit einem Plattenepithelkarzinom der oberen Luft- und Speisewege

Betreff: Erweiterte Auswertung der Studienergebnisse **zur Information**

Sehr geehrter Prof Richter,

wie mir Prof Strauch mitgeteilt hat, möchte er auf Grundlage der dargestellten Auswertung im Studienprotokoll (Bont V01F 23.07.2009, Kapitel 15) eine erweiterte Version des Mann-Whitney-U-Tests anwenden und noch eine zusätzliche Analyse mit den bestehenden Daten vornehmen, über die ich Sie mit den beiden angehängten Dokumenten informieren möchte.
Über ein baldige schriftliche Kenntnisnahme wäre ich Ihnen sehr dankbar.

Viele Grüße und Dank im Voraus,

S. Harnisch

Susanne Harnisch

Bankverbindung:
Sparkasse Marburg-Biedenkopf
Kto.-Nr. 130 280 01, BLZ 533 500 00

Anlage:

Der_van-Elteren-Test.doc
Sekundäranalyse_ohne_Baseline.doc

Der van-Elteren-Test

Der van-Elteren-Test ist eine Erweiterung des Mann-Whitney-U-Tests, die es gestattet, einen möglichen systematischen Unterschied zwischen der linken und der rechten Drüse, der unabhängig von der behandelten Seite besteht, auszugleichen. In unserem Falle soll auf einen Unterschied des Behandlungseffekts zwischen BoNT/A und kombinierter Gabe von BoNT/A-B getestet werden. Die beiden Behandlungen wurden in zwei Seiten bzw. Schichten (Strata) appliziert: links und rechts. Sollten nun systematische Unterschiede zwischen linker und rechter Drüse bestehen, was wir nicht ausschließen können, so kann dies zu einem Verlust an Trennschärfe (Power) des Mann-Whitney-U-Tests führen.

Der van-Elteren-Test kombiniert stratumsspezifische Teststatistiken eines Mann-Whitney-U-Tests zu einer kombinierten Teststatistik und gewichtet die stratumsspezifischen Beiträge umgekehrt proportional zur Stratumsgröße. Der Test betrachtet also zunächst nur die Unterschiede des Behandlungseffekts einer Seite, z.B. links, und berechnet die U-Statistik für diese Seite. Anschließend wird mit der zweiten Seite, in unserem Beispiel rechts, ebenso verfahren. Es wird also separat in beiden Seiten auf einen Unterschied bezüglich des Behandlungseffekts zwischen BoNT/A und BoNT/A-B getestet. Dies kann schon bei geringen systematischen Unterschieden der beiden Drüsen zu einer erhöhten Trennschärfe des van-Elteren-Tests im Vergleich zum Mann-Whitney-U-Test führen. Unter den gleichen Voraussetzungen wie für den Mann-Whitney-U-Test hält der van-Elteren-Test das angegebene Fehlerniveau der 1. Art ein. Die Nullhypothese für jedes Stratum (links, rechts) lautet: Kein Unterschied im Behandlungseffekt zwischen BoNT/A und -B. Die Alternativhypothese für jedes Stratum lautet: Unterschied im Behandlungseffekt zwischen BoNT/A und -B ungleich Null; zweiseitiges Testproblem. Die Hypothesen bezogen auf das einzelne Stratum entsprechen also genau denen des Mann-Whitney-U-Tests. Die kombinierte Alternativhypothese eines van-Elteren-Tests lautet: Unterschied im Behandlungseffekt zwischen BoNT/A und -B ungleich Null über alle Strata hinweg.

Bei Anwendung des van-Elteren-Tests werden weder eine andere Null- noch eine andere Alternativhypothese als beim Mann-Whitney-U-Test angenommen. Die Fragestellung und die Interpretation der Ergebnisse bleiben dieselben. Man ist lediglich in der Lage, für zusätzliche Varianz durch einen möglichen systematischen Seiteneffekt der Speicheldrüsen zu korrigieren, um so einen Verlust an Trennschärfe zu vermeiden. Dies haben wir durch eigene Computersimulationen bestätigen können.

Literatur:
van Elteren, P. H. (1960), „On the Combination of Independent Two Sample Tests of Wilcoxon," *Bulletin of the Institute of International Statistics*, 37, 351–361.

Analyse der Seitendifferenz des Uptakes und der Ejektionsfraktion ohne Baseline-Adjustierung

In dieser Sekundäranalyse wollen wir den Effekt und die Sinnhaftigkeit einer Adjustierung mit Baseline-Werten für diese Art von klinischer Studie feststellen. Es sollen alle vorgenannten Tests im Rahmen einer Sekundäranalyse lediglich unter Verwendung der Seitendifferenz 4 Monate nach der Behandlung, also ohne Berücksichtigung der Seitendifferenz vor der Behandlung (Baseline), durchgeführt werden. Konkret wird (i) mit dem Wilcoxon-Vorzeichen-Rangtest geprüft, ob die Uptake-Seitendifferenz 4 Monate nach der Behandlung innerhalb jeder der beiden Dosisgruppen (BoNT/A sowie BoNT/A und -B, je 6 Patienten) signifikant von Null abweicht, (ii) mit dem van-Elteren-Test geprüft, ob sich die Uptake-Seitendifferenz 4 Monate nach der Behandlung zwischen den beiden Dosisgruppen signifikant unterscheidet, und (iii) werden dieselben Analysen wie unter (i) und (ii) mit der Seitendifferenz der Ejektionsfraktion nach Gabe eines Reizgetränks 4 Monate nach der Behandlung, d.h. ohne Baseline-Adjustierung, durchgeführt.

Durch Nichtbeachten der Baseline-Werte soll es möglich sein, zumindest explorativ Behandlungseffekte zu erkennen, falls wider Erwarten die Seitendifferenz der behandelten minus unbehandelten Drüse nach der Behandlung nicht oder nur wenig mit der Differenz vor der Behandlung korreliert ist. Weiterhin wird als Sekundäranalyse eine lineare Regression der Seitendifferenz der behandelten minus unbehandelten Drüse 4 Monate nach der Behandlung mit der entsprechenden Differenz vor der Behandlung als Einflußvariable durchgeführt, und zwar sowohl bezüglich des Uptakes als auch der Ejektionsfraktion. Durch die Berechnung des Korrelationskoeffizienten zwischen den Differenzen vor bzw. nach der Behandlung sollen für zukünftige klinische Studien Informationen darüber gewonnen werden, ob es zweckmäßiger ist, die Seitendifferenz vor der Behandlung (Baseline) zu berücksichtigen oder nicht. Bei einem Korrelationskoeffizienten von unter 0.5 wäre es in Zukunft sinnvoller, nicht für die Baseline-Differenz zu adjustieren. Die Ergebnisse aller vorgenannten Sekundäranalysen zur Wirksamkeit von BoNT werden nicht konfirmatorisch interpretiert, sondern sollen lediglich als Hinweise für zukünftige klinische Studien dienen.

Literatur:
S. Senn, (2002). Cross-Over Trials in Clinical Research, Second Edition (Wiley), S. 62 ff.
