# Supplementary material for: Safety and Efficacy of Botulinum Toxin to Preserve Gland Function after Radiotherapy in Patients with Head and Neck Cancer: A Prospective, Randomized, Placebo-Controlled, Double-Blinded Phase I Clinical Trial
Source: PLoS One. 2016 Mar 18;11(3):e0151316. doi: 10.1371/journal.pone.0151316 (PMC4798778; doi:10.1371/journal.pone.0151316)
Supplement: S3 Text — (PDF) [file pone.0151316.s006.pdf]

Funktionsszintigraphische Untersuchungen zur Überprüfung der radioprotektiven Wirksamkeit intraglandulär applizierten Botulinum-Toxins (BoNT) auf die Gl. submandibularis bei Patienten mit einem Plattenepithelkarzinom der oberen Luft- und Speisewege

Korrektur zur Prüfplanversion V01F 2009_07_23, als Anlage dieser Prüfplanversion künftig beigelegt.

Grund der Veränderung: Es handelt sich bei der Medikation um handelsübliche Markenware, die in der vorgeschriebenen Art und Weise verwendet wird, so dass auf eine Kennzeichnung gemäß GCP-V, §5 (8) der Studienware verzichtet wird.

9.8 (S. 26) Verpackung und Labelling der Studienmedikation und Vergleichsmedikation

Die Medikation wird als handelsübliche Markenware in der vorgeschriebenen Art und Weise verwendet, so dass auf eine Kennzeichnung gemäß GCP-V, §5 (8) der Studienware verzichtet wird.
Es handelt sich um eine doppelverblindete Studie, wobei der Arzt und der Patient nicht wissen, welche Medikation in welche Glandula Submandibularis gespritzt wird. Über die eindeutige KKS-interne Nummer, die sowohl auf dem Umkarton als auch auf den einzelnen Einzelinjektionen für den Arzt vermerkt ist, ist eine eindeutige Zuordung der Studienmedikation anhand der Dokumentation des Apothekers (s.oben) möglich und jederzeit nachvollziehbar und bei Bedarf überprüfbar.
Nach Applikation der entsprechenden Injektion muss der Prüfarzt mit seiner Unterschrift und Datum die Applikation auf dem angebrachten Label bestätigen. Dieses Label muss nach Rückgabe der verbrauchten Studienware im studienspezifischen Apothekenordner aufbewahrt werden. Die Vernichtung verbrauchter Studienware und Verpackungen ist vom zuständigen Apotheker auf dem Medikations-log (drug-accountability) mit Unterschrift und Datum zu dokumentieren.

Funktionsszintigraphische Untersuchungen zur Überprüfung der radioprotektiven Wirksamkeit intraglandulär applizierten Botulinum-Toxins (BoNT) auf die Gl. submandibularis bei Patienten mit einem Plattenepithelkarzinom der oberen Luft- und Speisewege

Ergänzungen auf Wusch des Bundesamtes für Strahleschutz zur Prüfplanversion V01F 2009_07_23, als Anlage dieser Prüfplanversion künftig beigelegt.

Einschlusskriterien: Prüfplan S.9 (Synopsis) und 7.4, S.18

Grund der Veränderung: Das Bundesamt für Strahlenschutz hat eine Ergänzung und eine Korrektur von 2 Einschlusskriterien gefordert. Beide Veränderungen sind rein formaler Natur und haben keine Auswirkung auf die vorherige Aussagekraft der Einschlusskriterien.

- Patienten mit einem Plattenepithelkarzinom der oberen Luft- und Speisewege, ***bei denen bei denen eine primäre konventionelle Radio-Chemotherapie ärztlich indiziert ist***
- …….
- Frauen im gebärfähigen Alter ab [~~18]~~ ***40*** Jahren müssen eine der folgenden Methoden zur Empfängnisverhütung anwenden: orales Kontrazeptivum und Intrauterinpessar (Spirale), seit mindestens einem Monat vor Studienbeginn, Barriere mit Spermizid (Kondom oder Diaphragma), Tubenligatur. Als Frauen im gebärfähigen Alter werden alle Frauen angesehen, deren letzte Menstruation nicht länger als 1 Jahr zurückliegt
- …..
