# Supplementary material for: Safety and Efficacy of Botulinum Toxin to Preserve Gland Function after Radiotherapy in Patients with Head and Neck Cancer: A Prospective, Randomized, Placebo-Controlled, Double-Blinded Phase I Clinical Trial
Source: PLoS One. 2016 Mar 18;11(3):e0151316. doi: 10.1371/journal.pone.0151316 (PMC4798778; doi:10.1371/journal.pone.0151316)
Supplement: S4 Text — (PDF) [file pone.0151316.s007.pdf]

Radioprotektive Wirksamkeit von BoNT A/B

Case Report Form

Funktionsszintigraphische Untersuchungen zur Überprüfung der radioaktiven Wirksamkeit intraglandulär applizierten Botulinum-Toxins (BoNT) aauf die Gl. submandibularis bei Patienten mit Plattenepithelkarzinom der oberen Luft- und Speisewege

Pat.-ID-Nr.: ☐ **KKS-interne Nummer** ☐

Radioprotektive Wirksamkeit von BoNT A/B

Pat.-ID-Nr.: [ ]

Screening

Datum [ ] **(TT/MM/JJJJ)**

Einschlusskriterien

(Probanden dürfen nur eingeschlossen werden, wenn alle Einschlusskriterien mit JA beantwortet wurden!)

Patienten mit einem Plattenepithelkarzinom der oberen Luft- und Speisewege	[▽]
Alter >= 40 Jahre	[▽]
Schriftliche Einwilligung zur Teilnahme an der Studie	[▽]
Kognitive und physische Fähigkeit des Probanden, den Versuchsablauf zu verstehen und einzuhalten	[▽]
Frauen im gebärfähigen Alter ab 18 Jahren müssen eine der folgenden Methoden zur Empfängnisverhütung anwenden: orales Kontrazeptivum und Intrauterinpessar (Spirale), seit mindestens einem Monat vor Studienbeginn, Barriere mit Spermizid (Kondom oder Diaphragma), Tubenligatur. Als Frauen im gebährfähigen Alter werden alle Frauen angesehen, deren letzte Menstruation nicht länger als 1 Jahr zurückliegt	[▽]
Keine Funktionseinschränkungen und Seiten-Unterschiede im Rahmen der initialen Speicheldrüsenfunktionsszintigraphie	[▽]

Radioprotektive Wirksamkeit von BoNT A/B

Pat.-ID-Nr.:

Ausschlusskriterien

(Patienten dürfen nur eingeschlossen werden, wenn alle Ausschlusskriterien mit NEIN beantwortet wurden!)

Z.n. Exstirpation der GL. submandibularis

Anamnetisch bekannte Erkrankungen der Speicheldrüsen

Regelmässige Einnahme von Medikamenten, die die Funktion der Speicheldrüsen beeinflussen

Z.n. vorheriger Bestrahlung der Halsweichteile

Schwangerschaft

Stillzeit

Irenat-Therapie

Überempfindlichkeit gegenüber Botulinum-Toxin oder einem der sonstigen Bestandteile

Radioprotektive Wirksamkeit von BoNT A/B

Pat.-ID-Nr.: [ ]

1. Demographische Daten

Alter [ ] Jahre

Geschlecht:
- ◎ männlich
- ◎ weiblich

2. Tumordaten

Datum der histologischen Diagnose [ ] **(TT/MM/JJJJ)**

Lokalisation:

Mundhöhle [▽] Nasopharynx [▽] Oropharynx [▽]

Hypopharynx [▽] Larynx [▽]

andere Lokalisation [▽] [...]

Dignität

Plattenepithelkarzinom [▽]

andere Dignität [▽] [...]

Differenzierungsgrad [▽]

TNM-Klassifikation

◎ T1	◎ N1	◎ M0
◎ T2	◎ N2a	◎ M1
◎ T3	◎ N2b	
◎ T4	◎ N2c	
	◎ N3	

Tumor Stadium [▽]

Radioprotektive Wirksamkeit von BoNT A/B

Pat.-ID-Nr.: [ ]

3. Initiale Szintigraphie

Datum der Szintigraphie [ ] **(TT/MM/JJJJ)**

Ergebnis der Szintigraphie

Uptake der rechten Drüse		%
Uptake der linken Drüse		%
Uptake Unterschied rechts/links		%
Ejektionsfraktion nach Zitrone rechte Drüse		%
Ejektionsfraktion nach Zitrone linke Drüse		%
Unterschied Ejektionsfraktion nach Zitrone rechts/links		%

Radioprotektive Wirksamkeit von BoNT A/B

Pat.-ID-Nr.:

Studienbehandlung

5. Medikamentenapplikation (2 Wochen vor Bestrahlung)

Datum der Applikation ______ **(TT/MM/JJJJ)**

6. Strahlentherapie

Woche	Datum der Strahlentherapie (TT/MM/JJJJ)	Verabreichte Strahlendosis (Gy)
1		
2		
3		
4		
5		
6		

7. Chemotherapie

Woche	Datum der Chemotherapie (TT/MM/JJJJ)	Verabreichtes Medikament	Verabreichte Dosis (absolut)
1		...	
2		...	
3		...	
4		...	
5		...	
6		...	

Radioprotektive Wirksamkeit von BoNT A/B

Pat.-ID-Nr.: ☐

7. Kontroll-Szintigraphie (4 Monate nach Strahlentherapie)

Datum der Szintigraphie ☐ **(TT/MM/JJJJ)**

Ergebnis der Szintigraphie

Uptake der rechten Drüse	☐	%
Uptake der linken Drüse	☐	%
Uptake-Unterschied rechts/links	☐	%
Ejektionsfraktion nach Zitrone rechte Drüse	☐	%
Ejektionsfraktion nach Zitrone linke Drüse	☐	%
Unterschied Ejektionsfraktion nach Zitrone rechts/links	☐	%

Radioprotektive Wirksamkeit von BoNT A/B

Pat.-ID-Nr.: [ ]

Studienende

Datum: [ ] **(TT/MM/JJJJ)**

Art des Studienendes:

Studienende wie geplant [▽]

Abbruch wegen AE [▽] wenn ja, bitte AE Nr. angeben [ ]

Abbruch wegen SAE [▽] wenn ja, bitte SAE Nr. angeben [ ]

Mangelnde Compliance [▽]

Einwilligung zurückgezogen [▽]

wenn ja, dürfen witere Daten erhoben werden [▽]

Tod [▽] wenn ja, Datum [ ]

Todesursache [▽]

Andere Todesursache, bitte definieren:

[...]

Anderer Abbruchgrund [▽]

wenn ja, bitte definieren:

[...]

Radioprotektive Wirksamkeit von BoNT A/B

Pat.-ID-Nr.:

Begleitmedikation

Nr	Medikament (Generic Name)	Indikation	Von	Bis	Ongoing	AE relevant
	...	...				
	...	...				
	...	...				
	...	...				
	...	...				
	...	...				
	...	...				
	...	...				
	...	...				
	...	...				

Bitte überprüfen Sie, ob es sich bei der Indikation um ein neu aufgetretenes Adverse Event handelt und dokumentiren Sie dies gegebenenfalls im Formular Adverse Event

Radioprotektive Wirksamkeit von BoNT A/B

Pat.-ID-Nr.:

Adverse Event

Advese Event Description:

Serious: 0 = NO; 1 = YES --> If YES, please send SAE report form.

Intensity: 1 = mild; 2 = moderate; 3 = severe

Therapy of event: 0 = NO; 1 = Medication; 2 = Other; 3 = Medication + Other
Medication has to be documented in the Concommitant Medication Form. Other has to be documented as an Comment.

Outcome: 1 = recovered/ resolved; 2 = recovering/ resolving; 3 = not recovered/ not resolved; 4 = recovered/ resolved with sequale; 5 = fatal; 6 = unknown

Relation of event to Trial Medication: 1 = related; 2 = probably related; 3 = possibly related; 4 = unlikely; 5 = not related; 6 = not assessable

Action taken with Trial Medication: 1 = drug withdrawn; 2 = dose reduced; 3 = dose increased; 4 = dose not changed; 5 = unknown; 6 = not applicable
Interruption of medication has to be documented as two events (1. Event: dose reduced; 2. Event: dose increased)

AE No	Event	Start Date	End Date	Ongoing	Serious	Intensity	Therapy of Event
	...						
	...						
	...						
	...						
	...						
	...						
	...						
	...						

Comment: ◎ YES ◎ NO

Comment 1: (please refer to AE No.) ...

Comment 2: ...

Comment 3: ...
