# Supplementary material for: Safety and Efficacy of Botulinum Toxin to Preserve Gland Function after Radiotherapy in Patients with Head and Neck Cancer: A Prospective, Randomized, Placebo-Controlled, Double-Blinded Phase I Clinical Trial
Source: PLoS One. 2016 Mar 18;11(3):e0151316. doi: 10.1371/journal.pone.0151316 (PMC4798778; doi:10.1371/journal.pone.0151316)
Supplement: S6 Text — (PDF) [file pone.0151316.s009.pdf]

Supporting Information S10: Raw scintigraphic data.

Raw data of scintigraphic measurements at baseline, i.e., before radiotherapy (left part of the table) and after radiotherapy (right part of the table) are shown. For each patient, the individual ID number, the dose group (BoNT/A or BoNT/A-B), and information about the verum-treated gland (right or left) are given. After application of ^{99m}Tc-pertechnetate, i.e., the radionuclide, net counts for each gland (right and left) were calculated by subtracting the background activity (supraclavicular area) from i) the maximum counts before stimulation with lemon juice and ii) from the minimum counts after stimulation.
The uptake for each side was calculated according to:

$$\frac{(\text{Net maximum counts before stimulation} \times c)}{\text{Total activity of radionuclide [MBq]}},$$

where $c = 0.0001553$ refers to a collimator-specific constant.

The salivary excretion fraction (SEF) was calculated according to:

$$\frac{(\text{Net maximum counts before stimulation - Net minimum counts after stimulation})}{\text{Net maximum counts before stimulation}}.$$

Uptakes and SEFs are given as proportions; no conversion to percentages has been applied.
